# Development of New Live-Attenuated Vaccine Candidates Lacking Antibody-Dependent Enhancement (ADE) Against Dengue

**DOI:** 10.3390/vaccines13050532

**Published:** 2025-05-16

**Authors:** Brandon E. K. Tan, Seng Kong Tham, Chit Laa Poh

**Affiliations:** 1 ALPS Global Holding Berhad, The Icon, East Wing Tower Level 18-01 & Level 18-02, No. 1 Jalan 1/68F, Off Jalan Tun Razak, Kuala Lumpur 50400, Malaysia; brtek96@gmail.com (B.E.K.T.); drtham@alpsmedical.com (S.K.T.); 2 Nilai University, No.1, Persiaran Universiti, Putra Nilai, Bandar Baru Nilai, Nilai 71800, Malaysia

**Keywords:** dengue, live-attenuated vaccine, mutagenesis, attenuation, amino acid residue, antibody-dependent enhancement

## Abstract

Dengue virus (DENV) threatens public health, especially in regions with tropical and subtropical climates. In 2024, the World Health Organisation reported 3.4 million confirmed dengue cases, with 16,000 severe cases and 3000 dengue-associated fatalities. The first licensed dengue vaccine, CYD-TDV (Dengvaxia^®^,Sanofi-Pasteur, Paris, France), is recommended by the WHO only for individuals aged 9–45 years with a prior history of dengue infection. However, being vaccinated with Dengvaxia^®^ increases the risk of developing severe dengue infections in seronegative individuals. Recently, a second licensed dengue vaccine, Qdenga^®^,Takeda, Singen, Germany), was approved and recommended by the WHO to be administered only in highly dengue-endemic countries, as it was not shown to elicit a robust immune response against DENV-3 and DENV-4 serotypes in dengue seronegative individuals. Due to an imbalance in immune response against all four DENV serotypes, there is a higher risk of developing the antibody-dependent enhancement (ADE) effect, which could lead to severe dengue. This review has identified mutations throughout the DENV genome that were demonstrated to attenuate the virulence of DENV in either in vitro or in vivo studies. Several amino acid residues within the DENV prM and E proteins were identified to play important roles in ADE and modifying these ADE-linked residues is important in the rational design of novel live-attenuated dengue vaccine candidates. This review provides current insights to guide the development of a novel live-attenuated tetravalent dengue vaccine candidate that is effective against all DENV serotypes and safe from ADE. The efficacy and safety of the live-attenuated vaccine candidate should be further validated in in vivo studies.

## 1. Introduction

Dengue virus (DENV), a single-stranded positive-sense RNA virus of the *Flaviviridae* family is known to be responsible for the most rapidly spreading mosquito-borne disease globally [1]. Approximately half of the current world population resides in tropical and subtropical regions with a high risk of dengue transmission. The World Health Organisation (WHO) reported that the global burden of dengue has increased 30-fold over the past 50 years, mainly due to rapid urbanisation, accelerated geographical expansion, exponential population growth, and unprecedented climate change. A positive correlation between rising global temperature and increased dengue incidence has suggested that unprecedented climate change could accelerate the global spread of dengue [2]. Dengue is now considered endemic in more than 100 countries across South East Asia, South Asia, the Americas, the Western Pacific, and Africa, with Asia alone intriguingly accounting for 70% of the global dengue burden [3]. A comprehensive study utilising data obtained from the Global Burden of Disease Study 2019 (GBD 2019) to analyse dengue transmission patterns estimated a significant rise in dengue fever incidence from 1990 to 2019, with the highest incidence rates being present in Oceania, followed by South Asia and South East Asia [4]. While dengue is traditionally known to be widespread in tropical and subtropical regions, the unusual rate of climate change would highly facilitate the spread of dengue Aedes vectors to continental Europe [5].

In 2009, the WHO reported up to 100 million dengue infections occurring yearly, with around 500,000 cases of severe dengue infections and 25,000 dengue-associated deaths [6]. Later in 2013, a study utilising cartographic approaches combined with dengue cohort and dengue incidence population records suggested that the actual number of dengue infections is in fact as high as 390 million annually, nearly three times the dengue burden estimate provided by the WHO official report in 2009 [3,6]. This glaring discrepancy further addresses the growing challenge to assess and manage the global impact of dengue accurately. In 2024 alone, the WHO reported over 7.6 million dengue cases occurring globally, including 3.4 million confirmed dengue cases, which include 16,000 severe dengue and 3000 dengue-related deaths [7]. Over the past five years, there has been a significant increase in dengue cases being reported globally, where the rise has been apparent in the Americas. As of April 2024, dengue cases in the Americas were reported to have exceeded 7 million, superseding the annual high of 4.6 million cases in the previous year.

In this review, we discussed the molecular determinants located throughout the DENV genome that are likely to be strongly associated with virulence activity in both in vitro and in vivo studies. Mutations that have been incorporated into the Sanofi Pasteur CYD-TDV (Dengvaxia^®^), Takeda TAK-003 (Qdenga^®^), NIAID TV003/TV005, and the Butantan-DV live-attenuated dengue vaccines will not be discussed in this review. Also, mutations that have been demonstrated to lead to a replication-defective phenotype and inherently unstable genotype that could lead to reversion to wild type will be excluded from being incorporated into a proposed new live-attenuated dengue vaccine candidate. We seek to provide some current up-to-date insights that will contribute to the novel development of a live-attenuated dengue vaccine for clinical applications in low- and middle-income countries.

## 2. DENV Clinical Manifestations

DENV is known to be mainly transmitted by the bite of infected female mosquitoes from the *Aedes* genus, predominantly *Aedes aegypti* and *Aedes albopictus*, via horizontal transmission [8]. From this process, the mosquito becomes infected upon the ingestion of blood from a virally infected host [9]. Following an extrinsic 10-day incubation period, DENV will travel from the mosquito’s intestinal tract to the salivary glands. The infected mosquito will then proceed to bite another person, transmitting DENV in the process [9,10]. DENV has four serotypes: DENV-1, DENV-2, DENV-3, and DENV-4 which co-circulate globally, and they share approximately 65% of their genomes [9]. Humans are regularly exposed to cross-species transmission of viruses, which leads to the sustained circulation of all DENV-1 to DENV-4 lineages today [11]. Within an incubation period of 3 to 7 days following the bite of a DENV-infected mosquito, the disease progresses through three distinct phases: an initial febrile phase, a critical phase during defervescence, and a spontaneous recovery phase [12]. Approximately 75% of DENV cases are asymptomatic, whilst symptomatic patients normally present classical dengue fever (DF), a self-limiting but debilitating illness characterised by high fever, vomiting, and rashes during the febrile phase [12,13]. However, a small subset of patients may develop severe, life-threatening dengue haemorrhagic fever (DHF), which is marked by vascular leakage of blood plasma, thrombocytopenia, and, on some occasions, can lead to dengue shock syndrome (DSS), which is a critical state involving haemorrhaging and circulatory failure. Following the critical phase, patients typically enter the recovery phase, in which adult patients may experience profound fatigue that will persist for several weeks after recovery [12].

## 3. Antibody-Dependent Enhancement (ADE) in Dengue Infections

When a person is infected with a DENV serotype strain (e.g., DENV-1), it triggers the production of serotype-specific homotypic antibodies that typically provide life-long immunity against subsequent infections by the same serotype [14]. However, these antibodies demonstrate limited cross-protective efficacy against heterologous DENV serotypes (e.g., DENV-2, DENV-3, DENV-4). Thus, following a secondary infection with any of the other heterologous serotypes, patients often experience more severe dengue clinical symptoms. This underlying phenomenon is known as antibody-dependent enhancement (ADE), which has been comprehensively studied in various viruses, including DENV [15,16,17,18]. ADE can be characterised by two different mechanisms, antibody-mediated replication (extrinsic ADE) and enhanced immune activation (intrinsic ADE) [19]. Extrinsic ADE mainly occurs in DENV infection in the presence of sub-neutralising or heterotypic non-neutralising antibodies, forming a virus–antibody complex [14,20]. This newly generated virus–antibody complex facilitates the secondary infection of phagocytic cells expressing Fc gamma receptors (FcγR). The FcγR will bind with the Fc region of the non-neutralising antibody while the antibody’s Fab region will bind with the viral antigen, resulting in the entry of the virus–antibody complex into the immune cell [21]. This culminates in enhancing viral replication and spread, which can potentially lead to an increased risk of severe dengue infection. On the contrary, intrinsic ADE is primarily observed in respiratory viruses such as measles, and this involves the activation of the complement pathway by the virus–antibody complex [19]. Following activation of the complement pathway, enhancement of disease severity is driven by the release of excess pro-inflammatory cytokines and complements that will be deposited in airway tissues, potentially causing an obstruction.

## 4. DENV Genome Structure

**Figure 1:**
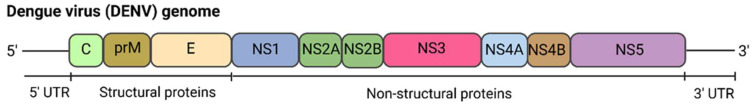
The dengue virus (DENV) genome shows the genes that encode structural and non-structural proteins. DENV structural proteins consist of the C, prM, and E proteins. DENV non-structural proteins consist of seven non-structural proteins (NS1, NS2A, NS2B, NS3, NS4A, NS4B, and NS5).

DENV is a small, well-studied enveloped virus harbouring a single-stranded positive-sense RNA genome encapsulated in an icosahedral nucleocapsid covered by the lipid bilayer [22]. The 11kb long (+) RNA genome encodes a single open reading frame (ORF), with the 5′ UTR and 3′ UTR regions flanking the ORF. The ORF encodes a single viral polyprotein that comprises 3400 amino acids. Both co- and post-translational processing of this single viral polyprotein leads to the generation of ten mature DENV proteins, three structural and seven non-structural proteins (Figure 1) [23,24]. The structural proteins are the capsid (C), pre-membrane protein (prM), and envelope (E), which are mainly involved in nucleocapsid formation, envelopment, and the maturation of the progeny virions [25]. On the other hand, the non-structural proteins are arranged in the order of NS1, NS2A, NS2B, NS3, NS4A, NS4B, and NS5 and they are involved in multiple roles of the DENV life cycle in terms of viral RNA replication and infectious virus production. The DENV genome features a m7GpppAmpN2 cap modification at its 5′ untranslated region (5′ UTR), and the 3′ untranslated region (3′ UTR) forms a characteristic stem-loop secondary structure [26].

Evidence of genetic variability among the four DENV serotypes was established via nucleotide sequencing and enzymatic amplification of RNA genome approaches [27]. Comparative analyses of multiple dengue viruses and the subsequent construction of phylogenetic trees have helped to establish the evolutionary relationships between strains, both within a single serotype and across different serotypes [27,28]. Consequently, the term ‘genotype’ is now commonly utilised to categorise genetically distinct variant groups within each of the DENV serotypes. Based on nucleotide sequence comparisons, researchers have widely proposed hypotheses regarding the origin, nature, and evolution of the different lineages [29]. Genetic analysis of strains with differing pathogenicity has identified variations in nucleotide and polyprotein sequences, indicating their potential contribution to virulence [30]. Thus, the next step involves searching for strategies to elucidate the differences in the pathogenesis of different genotypes and to assess the role of viral virulence in the specific disease [27].

## 5. Current Live-Attenuated Dengue Vaccines

**Figure 2:**
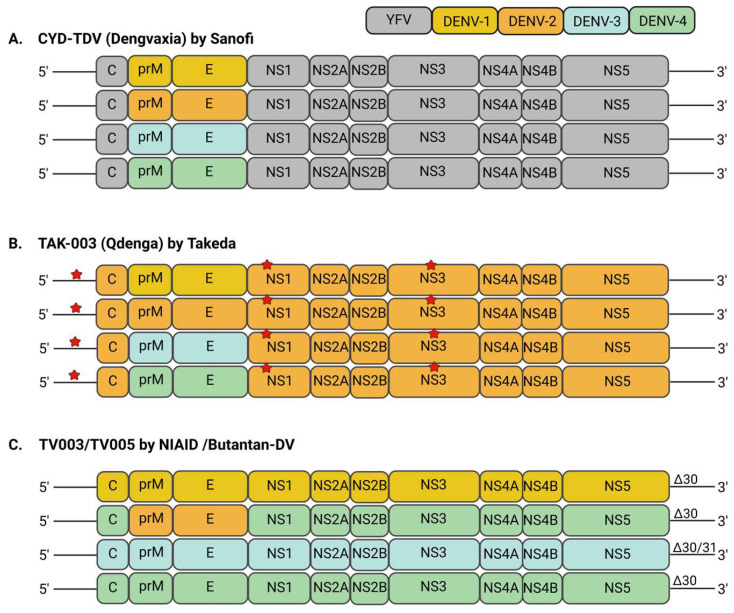
Schematic diagrams of the tetravalent live-attenuated dengue vaccines. (**A**) CYD-TDV (Dengvaxia^®^) by Sanofi comprised the YFV-17D backbone vector (grey) with the respective prM and E genes from the DENV-1 (brown), DENV-2 (orange), DENV-3 (blue), and DENV-4 (green) genomes. (**B**) TAK-003 (Qdenga^®^) by Takeda comprised the DENV-2 genome from an attenuated DENV-2 strain and three YFV-DENV chimeric viruses expressing the prM and E genes of DENV-1, DENV-3, and DENV-4, respectively. Red star indicated the individual attenuating mutation located within the 5′ untranslated region (UTR) (C57ntT), NS1 protein (G53D), and the NS3 (E250V). (**C**) TV003/TV005 by NIAID comprised three full-length wild type viruses and one chimeric virus, in which the prM and E genes of DENV-4 were replaced by those from DENV-2. The viruses within the TV003/TV005 vaccines were attenuated with the introduction of a 30-nt deletion (Δ30) and an additional 31-nt deletion (Δ31) located upstream of the Δ30. YFV (Grey), DENV-1 (brown), DENV-2 (orange), DENV-3 (blue), and DENV-4 (green).

Despite dengue being present for many centuries, the earliest efforts to develop a DENV vaccine began in the 1920s [31]. As time progresses, significant advances in tissue culture and recombinant DNA technology have massively contributed to progress in DENV vaccine development [32]. However, multiple challenges have severely impeded the development of a successful DENV vaccine. These setbacks are contributed to by the presence of four antigenically distinct serotypes, incompletely defined immunopathogenic responses after vaccination or natural infection, and a lack of established and validated animal models for dengue [33,34,35]. Following many decades of persistent research, the world’s first dengue vaccine, manufactured by Sanofi Pasteur, Dengvaxia^®^ or CYD-TDV, was finally approved and licensed in late 2015 [36]. CYD-TDV is a tetravalent live-attenuated recombinant vaccine in which the structural genes, prM and E genes of the YFV-17D virus vector were replaced by structural genes from each of the four DENV serotypes (Figure 2A) [37]. CYD-TDV is administered in a three-dose schedule with a 6-month interval between doses for individuals aged 9–45 years and residing in highly dengue-endemic areas. It was first licensed in Mexico in 2015 and as of December 2017, was licensed in 19 countries, including Brazil, Philippines, Singapore, Indonesia and Thailand. In April 2016, both the Philippines and Brazil were the first countries to incorporate a national CYD-TDV immunisation campaign against dengue [38]. A related study estimated that 5 years after Dengvaxia^®^ vaccination, the number of severe dengue cases within the vaccinee population would decrease by approximately 70% [39]. In December 2018, Dengvaxia^®^ acquired European Union marketing authorisation approval for administration to people aged 6–45 years with a prior history of dengue infection.

In a phase III clinical trial conducted in five highly dengue-endemic Latin American countries, the overall CYD-TDV vaccine efficacy in healthy children aged 9–16 years was 64.7% [40,41]. Intriguingly, CYD-TDV vaccine protections were much higher against DENV-3 and DENV-4 at 74% and 77%, than against DENV-1 and DENV-2 at 50.3% and 42.3%, respectively. Unfortunately, in late 2017, the CYD-TDV national immunisation campaign in the Philippines was immediately discontinued following the deaths of 14 children previously vaccinated with CYD-TDV a few years ago [38]. In a subsequent study funded by Sanofi Pasteur, concerns were further raised about the dengue serostatus of the CYD-TDV vaccinees. In this study, 3 years after receiving the CYD-TDV vaccine, there was an increasing trend in hospitalisation risk for the vaccinees, especially among the dengue-seronegative vaccinees, suggesting that the vaccine was far more suitable for dengue-seropositive vaccinees in terms of superior efficacy and beneficial vaccine effect [42,43]. In September 2018, the WHO highly recommended that dengue-seronegative individuals should not be vaccinated with CYD-TDV as there is a possibility for an increased risk of developing severe dengue [44]. Thus, countries were advised to reconsider the introduction of CYD-TDV in their national immunisation programmes.

A second tetravalent live-attenuated dengue vaccine, TAK-003 (Qdenga^®^), developed by Takeda, has recently been licensed and recommended by the WHO in highly dengue-endemic countries globally [45]. The WHO recommended the administration of TAK-003 in children (6–16 years of age) who are residing in countries with high dengue transmission intensity. TAK-003 is composed of the live-attenuated DENV-2 PDK-53 strain (TDV-2) serving as the genome backbone, with its prM and E genes replaced by those from DENV-1 16007 (TDV-1), DENV-3 16562 (TDV-3), and DENV-4 1036 (TDV-4) (Figure 2B) [46]. The PDK-53 strain was originally developed by serially passaging the DENV-2 16681 virus 53 times in primary dog kidney (PDK) cells [47]. The attenuated PDK-53 strain displayed several distinct characteristics, including replication efficiency, attenuated neurovirulence, higher genetic diversity, and temperature sensitivity in comparison to its parental DENV-2 16681 strain [47,48]. Additionally, these features were predominantly attributed to several attenuating mutations in the 5′ untranslated region (UTR) (C57ntT), non-structural 1 (NS1) protein (G53D), and non-structural 3 (NS3) protein (E250V) of the DENV genome. It has also been suggested that there was a risk of reversion of the DENV-2 PDK-53 to the wild type 16681 if both 5′ UTR and NS1 mutations were to revert concurrently [47].

To evaluate the long-term efficacy of the TAK-003 vaccine across several highly dengue-endemic countries, 20,071 healthy adolescents, aged 4–16 years, were administered either the TAK-003 or placebo in a two-dose schedule [49,50]. TAK-003 successfully demonstrated its sustainable efficacy and safety against symptomatic dengue over 4.5 years against all four DENV serotypes in dengue seropositive individuals but was only effective against DENV-1 and DENV-2 serotypes in dengue seronegative individuals [50]. Protective efficacy appeared to wane over time, but remained robust against hospitalised dengue-infected patients, prompting the need for a booster dose evaluation. The WHO does not recommend countries with low to moderate dengue transmission settings to utilise the TAK-003 vaccine as the efficacy risk profile for DENV-3 and DENV-4 serotypes in dengue-seronegative patients remains to be further elucidated [45].

## 6. Live-Attenuated Dengue Vaccines in Phase III Clinical Trials

The TV003/TV005 tetravalent live-attenuated dengue vaccine was developed by the National Institute of Allergy and Infectious Diseases (NIAID), United States, by creating attenuated DENV strains through the introduction of 30- and 31-nucleotide deletions in the 3′ UTR (Figure 2C) [51]. Six monovalent DENV vaccine candidates were assessed for efficacy in preclinical studies involving mice and non-human primates, but only four candidates were selected to represent each DENV serotype in the final tetravalent vaccine formulations [48]. These monovalent DENV vaccine candidates are rDEN1Δ30 (DENV-1), rDEN2/4Δ30 (DENV-2), rDEN3-3′D4Δ30 or rDEN3Δ30/31 (DENV-3) and rDEN4Δ30 or rDEN4Δ30-200,201 (DENV4) [52,53,54,55,56,57,58]. Both rDEN1Δ30 and rDEN4Δ30 were generated via the introduction of a 30-nucleotide deletion (Δ30) into the 3′ UTR of DENV-1 and DENV-4 genomes, respectively. After that, rDEN2/4Δ30 was created via the replacement of the prM and E genes of rDEN4Δ30, with the prM and E genes from DENV-2. Then, the rDEN3-3′D4Δ30 was generated by replacing the whole DENV-3 3′ UTR region with the 3′ UTR of rDEN4Δ30. Next, rDEN3Δ30/31 was engineered to harbour an additional 31-nucleotide deletion (Δ31) upstream of the Δ30 deletion mutation. Lastly, the rDEN4Δ30-200,201 was based on rDEN4Δ30 that harboured alanine substitutions at amino acid positions 200 and 201 of the NS5 protein.

**Figure 3:**
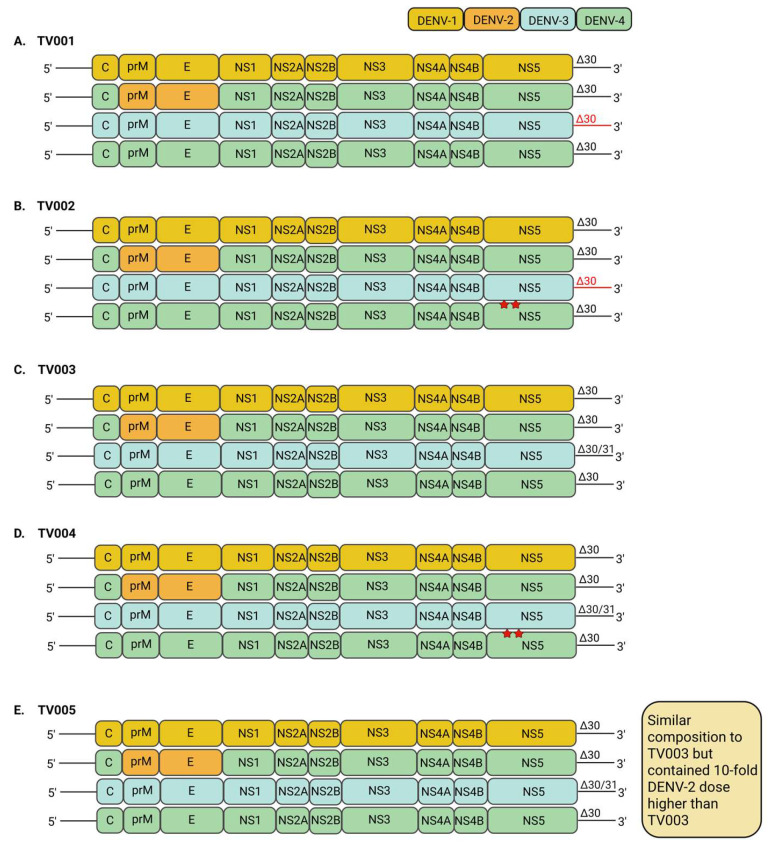
Schematic diagram of the tetravalent TV001-TV005 admixtures. (**A**) TV001 consisted of rDEN1Δ30, rDEN2/4Δ30, rDEN3-3′D4Δ30 and rDEN4Δ30. (**B**) TV002 included rDEN1Δ30, rDEN2/4Δ30, rDEN3-3′D4Δ30 and rDEN4Δ30-200,201. (**C**) TV003 contained rDEN1Δ30, rDEN2/4Δ30, rDEN3Δ30/31 and rDEN4Δ30. (**D**) TV004 was composed of rDEN1Δ30, rDEN2/4Δ30, rDEN3Δ30/31 and rDEN4Δ30-200,201. (**E**) TV005 had a similar composition to TV003, but TV005 contained a ten-fold higher dose of the DENV-2 dose component than TV003. The red star indicated the individual attenuating mutation within the DENV-4 NS5 at amino acid positions 200 and 201. Δ30/31 indicated the introduction of a 30-nt deletion in the 3′ UTR with an additional 31-nt deletion upstream of the Δ30. Δ30 (in red) indicated that the whole 3′ UTR of the DENV-3 was replaced by the 3′ UTR of the DENV-4 containing the Δ30 deletion (rDEN4Δ30). YFV (Grey), DENV-1 (brown), DENV-2 (orange), DENV-3 (blue), and DENV-4 (green).

Five tetravalent admixtures (TV001-TV005) were formulated differently, with the DENV-1 and DENV-2 monovalent components remaining the same across the five admixtures, while DENV-3 and DENV-4 monovalent components varied slightly (Figure 3). The TV001 was consisted of rDEN1Δ30, rDEN2/4Δ30, rDEN3-3′D4Δ30 and rDEN4Δ30 [48]. TV002 included rDEN1Δ30, rDEN2/4Δ30, rDEN3-3′D4Δ30 and rDEN4Δ30-200,201 while TV003 contained rDEN1Δ30, rDEN2/4Δ30, rDEN3Δ30/31 and rDEN4Δ30. TV004 was composed of rDEN1Δ30, rDEN2/4Δ30, rDEN3Δ30/31 and rDEN4Δ30-200,201. On the other hand, TV005 had a similar formulation to TV003 except that it contained a ten-fold higher dose of the DENV-2 component than in TV003 [59].

A single dose of TV003 or TV005 vaccine candidate was highly efficient in eliciting a balanced neutralising immune response against all four DENV serotypes, with a two-dose schedule within the 6-month interval between the doses [60]. TV005 with a ten-fold higher dose of the DENV-2 component displayed higher immunogenicity for DENV-2 in comparison to TV003, with 97% and 76% seroconversion induced by TV005 and TV003, respectively. Another TV003 vaccine dosing study was conducted to determine if a two-dose schedule within a 12-month interval between two doses could effectively improve the neutralising antibody titers [61]. Results showed that it did not significantly boost neutralising antibody titers to any of the DENV serotypes. Thus, a booster dose, regardless of the 6- or 12-month interval, was deemed unnecessary as it provided only minimal benefits.

The Butantan Institute in Brazil obtained a license for TV003 from the NIAID and proceeded to develop a vaccine that was analogous to TV003 but not identical, named the Butantan-DV vaccine [62]. The Butantan-DV was assessed in phase II clinical trials involving both dengue seropositive and seronegative participants in a two-dose schedule with a 6-month interval between the two doses. Participants administered the Butantan-DV were observed to experience minor rashes. A single dose of the Butantan-DV was shown to induce robust, balanced neutralising antibody responses against the four DENV serotypes, whilst a booster dose did not significantly improve the geometric mean titer (GMT) of neutralising antibodies. With the TV003, the Butantan-DV only requires a single dose to adequately confer protection against dengue [60,62]. An ongoing phase III clinical trial in Brazil with a 5-year follow-up period after the administration of the single-dose Butantan-DV to 16,235 dengue seropositive and seronegative individuals (aged between 2 and 59 years) was in its 2-year follow-up period [63]. The overall vaccine efficacy against any DENV serotype was 79.6%. The Butantan-DV vaccine efficacy was much higher in dengue seropositive individuals (89.2%) in comparison to the dengue seronegative individuals (73.6%). In terms of serotype-specific efficacy, vaccine efficacies against DENV-1 and DENV-2 were 89.6% and 69.6%, respectively. There were no observed efficacies against DENV-3 and DENV-4 cases in this follow-up period, so the vaccine efficacies against both DENV-3 and DENV-4 were undetermined. A single dose of the Butantan-DV demonstrated convincing safety profiles and was deemed efficacious in preventing symptomatic DENV-1 and DENV-2 infections, irrespective of the dengue serostatus within this two-year follow-up period.

## 7. 5′ Untranslated Region (5′ UTR)

The non-coding 5′ UTR of nearly all flaviviruses spans approximately 100 nucleotides in length and is located upstream of the capsid gene [64]. Six distinct elements are present in the 5′ UTR, including the two highly conserved stem-loop structures (SLA and SLB), the 5′ upstream AUG region (5′ UAR), the 5′ downstream AUG region (5′-DAR), the capsid coding hairpin (cHP) and the 5′ cyclisation sequence (5′ CS) [65]. The SLA and SLB regions are around 70 and 30 nucleotides, respectively [66]. A poly(U) sequence separates the SLA and SLB to enable the proper functioning of these regions, as they are essential for RNA synthesis. The SLB, which is located nearest to the ORF start codon, includes the 5′-UAR sequence necessary for long-range RNA interactions during viral genome replication [67]. Highly conserved among flaviviruses, the cHP motif has been functionally characterised to be involved in start codon recognition and viral replication [66]. In short, all these elements are highly essential for viral RNA replication, translation, and pathogenesis in mammalian and mosquito cells.

Deletion of a series of consecutive nucleotides from position 82 to 87 within the 5′UTR (Δ82–87) of the DENV-4 genome generated viable infectious viruses with a marked reduction in viral RNA translation efficiency in comparison to the wild type DENV [68] (Table 1). The mutant progeny viruses (Δ82–87) produced distinctly smaller plaques in rhesus monkey LLC-MK2 epithelial cells but failed to produce plaques in C6/36 mosquito cells. Another study investigated the effects of introducing various deletions or point mutations specifically between nucleotides at positions 54 and 70 in the 5′ UTR [69] (Table 2). Mutants with multiple deletions or substitutions failed to produce viable viruses. However, a substitution mutation at nucleotide position 69 from A to T led to a lower mortality rate in mice but interestingly produced plaque sizes that were comparable to the wild type.

## 8. Capsid (C)

The capsid is a relatively small monomeric protein that spans approximately 100 amino acids, containing 26 basic and 3 acidic amino acids [70]. Structural studies via nuclear magnetic resonance (NMR) analysis successfully resolved the three-dimensional structure of the DENV C protein, which consists of four alpha helices (α1 to α4) [71]. A conserved internal hydrophobic region located between amino acids at positions 45 and 64 of the mature capsid protein has been shown to associate with the host ER membrane, possibly to facilitate virion entry into the host ER [72]. Located within the capsid-coding region, a highly conserved RNA hairpin structure (cHP) among DENV and West Nile virus (WNV) modulates translation start codon selection and is deemed essential for viral replication [73]. Abrogating the cHP and the first two AUG codons within the capsid coding region led to a significant reduction in viral replication to undetectable levels by plaque assays in HEP3 and C6/36 cells, despite lower levels of viral replication being observed in the second AUG codon mutant. Clyde and colleagues (2008) demonstrated that an abrogated cHP can be rescued by compensatory mutations that restabilised the cHP [74]. Two compensatory mutations, A132C and A204G, were identified, and both appeared to exhibit contrasting compensation activities. The A132C mutation rescued replication efficiency comparable to the wild type, whilst the A204G still retained a defect in viral replication.

When the α3 helix (amino acid positions 42–59) of the capsid protein was deleted, the resulting mutant viruses became completely attenuated [75]. Intriguingly, these mutated viruses exhibited reduced virulence in suckling mice, but in contrast, were found to elicit high levels of antibodies in adult mice. In a bid to investigate nuclear localisation signals, several double alanine substitutions were introduced into the DENV capsid protein at proposed nuclear localisation signal sequences that are highly conserved among DENV serotypes [76]. Mutant clones (K6A/K7A) and (K73A/K74A) successfully generated viable viruses with markedly reduced replication efficiency in comparison to the wild type in porcine kidney (PS) epithelial cells, Vero cells, and C6/36 cell lines. Despite the K6A/K7A mutant virus displaying reduced nuclear localisation in PS cells 72 h post-infection, there was no clear correlation between nuclear localisation and viral replication activity. DENV capsid protein has been shown to gradually accumulate around the surface of ER-derived organelles known as lipid droplets (LDs) in DENV-infected cells, and they are important for viral replication [77]. Substituting L with S at highly conserved position 54 within the hydrophobic cleft was sufficient to impede capsid protein accumulation on the surface of LDs. Although the L54S mutation altered capsid protein targeting the LDs and led to defects in infectious particle production, it did not impair viral RNA translation or synthesis. Despite the L54S mutation not being able to ideally attenuate the virus, it remains an important tool to study other uncharacterised residues within the capsid protein that might associate with the surface of LDs and influence viral replication.

## 9. Pre-Membrane/Membrane (prM/M)

The DENV prM glycoprotein constitutes 166 amino acids in length [78]. Following translation of the single DENV polyprotein from the ORF that is cleaved by cellular and host proteases to liberate the individual viral proteins, the mature membrane (M) protein is first produced as a precursor named the precursor-membrane glycoprotein (prM). The prM protein comprises a N-terminal domain that includes a pre-domain (pr) peptide (found in immature virus), M-domain containing a furin cleavage site, an α-helical stem structure (MH), and two transmembrane helices, MT1 and MT2, within the C-terminal transmembrane domain [79]. The pr peptide’s β-barrel configuration shields the E protein’s fusion loop, thereby preventing premature membrane fusion in immature virions [78]. The fusion loop of the E protein is concealed by the β-barrel structure of the pr peptide, preventing the immature virus from fusing with the host membrane. The low pH conditions within the trans-Golgi network trigger structural rearrangement of the prM, allowing the accessibility of the furin cleavage site (amino acid position 91) to the host resident protease furin and enabling the cleavage of the prM protein [80]. During maturation, the host resident protease furin, cleaves the prM, liberating the N-terminal “pr” (amino acid positions 1–91), leaving behind the ectodomain (amino acid positions 92–130) and C-terminal transmembrane domain (amino acid positions 131 to 166) of the M protein [78]. Following proteolytic cleavage, the pr peptide remained non-covalently bound to the E protein fusion loop region, only dissociating from it when the mature virions encountered neutral pH in the extracellular milieu.

The construction of chimeric viruses incorporating the prM-E genes of DENV-1, -3 or -4 serotypes within the YF-17D live vector backbone led to the identification of two substitution mutations within the M protein [81]. A substitution mutation from histidine to arginine residue at position 39 of the M protein (M39), accompanied by two other mutations of the E protein within the chimeric YF/DENV-1 virus, led to a reduction in viraemia in rhesus monkeys when compared to the wild type DENV-1 virus. Additionally, a substitution mutation from alanine to threonine residue at position 43 of the M protein (M43) in the chimeric TF/DENV-4 virus, also demonstrated low levels of viraemia in comparison to the wild type DENV-4 virus. The histidine residue at M39 is only highly conserved among DENV-1, DENV-2 and DENV-3 and appears as an asparagine residue at M39 in DENV-4 and other flaviviruses. In contrast, the alanine at M43 is only present in DENV-4, whilst a threonine residue is present in DENV-1, DENV-2, and DENV-3. Pryor et al. (2004) identified several other amino acid residues, including M39, that are involved in maintaining the structural integrity of the M protein [82]. Mutations encoding amino acids that were either basic, non-polar, or uncharged polar were individually introduced to substitute the histidine residue at position 39. Substitution of the histidine residue with an uncharged polar glutamine residue showed a significant reduction in viral replication efficiency in comparison to the wild type. Pryor and colleagues (2004) also discovered that substitution of the histidine residue with an asparagine or arginine residue at position 39 resulted in a slight reduction (~1.7–2.2 log10) of virus titers when compared to the wild type. Interestingly, substitution of the histidine with non-polar amino acid residues like leucine and proline showed no viral replication activity, suggesting that a polar amino acid is always present at position 39. Nevertheless, the M39 residue has been identified as a critical residue that may be essential in the DENV life cycle.

In a mutagenesis study, alanine substitutions were introduced to nine highly conserved residues among the DENV serotypes within the MH domain (amino acid positions 112–131) to elucidate the role of the elusive MH domain in the DENV life cycle [83]. All the alanine mutants except S112A impaired the entry of replicon particles, as observed with more than ~1 log reduction in luciferase activity compared to the wild type. Upon quantifying the amounts of RNA in replication particles, three alanine mutants E124A, L128A, and R129A, displayed more than ~2 log reduction in the amounts of replicon RNA compared to the wild type, relatively consistent with the effect of these mutations impairing the assembly of replicon particles. A recent phylogenetic analysis of South Pacific Islands (SPI) DENV-2 isolates demonstrated that almost all Tonga DENV-2 strains (characterised by an arginine substitution at position 86 (H86R) in the prM protein), formed a distinct cluster from the other SPI DENV-2 strains [84]. The prM H68R mutation resulted in the attenuation of the mutant H86R virus as seen with the generation of small plaques and relatively low viral replication efficiency, in comparison to the regional DENV-2 strains. Intriguingly, the prM H86R mutation appeared to increase the ability of the Tonga DENV-2 strains to infect the midgut of the *Aedes aegypti* mosquitoes, which was strongly suggested by the authors as a strategic fitness trade-off to ensure continuous effective transmission, compromising viral replicative fitness in the process. Of note, the histidine residue at position 86 of the prM is highly conserved across all DENV serotypes except in DENV-4, which interestingly has an arginine residue at this position.

## 10. Envelope (E)

The envelope (E) protein is a key surface glycoprotein belonging to class II viral fusion proteins that plays a crucial role in viral internalisation into host cells via receptor-mediated endocytosis [85]. The 400 amino acid-long DENV E protein consists of an extensive β-sheet-rich ectodomain, a membrane-adjacent stem region, and a transmembrane anchoring region consisting of two helical segments at its C-terminus [86,87]. The ectodomain is structurally folded into three distinct β-barrel domains, domain I (E-DI), domain II (E-DII), and domain III (E-DIII) [88]. E-DI adopts a β-barrel-like structure and is flanked on one side by an elongated, finger-like structure. E-DII contains a hydrophobic fusion peptide loop (amino acid positions 98–111) at its distal end that enables attachment of the virus to the host cell. On the opposite side of E-DI lies an immunoglobulin (Ig)-like domain, E-DIII, which is believed to possess host cell receptor binding properties and is connected to E-DI via a single polypeptide linker. Antibodies exhibiting strong neutralisation activities have been thoroughly mapped to epitopes located within E-DIII [89]. The soluble E-DIII has been widely utilised to impede infection of cells by a whole virus, suggesting that the E-DIII domain possesses receptor-ligand epitopes that enable the elicitation of virus-specific neutralising antibodies.

Huang and colleagues (2010) conducted a comprehensive study investigating the roles of specific amino acids within the fusion peptide of the DENV-2 E protein in virus-mediated membrane fusion and viral replication [90]. A site-directed mutagenesis approach was utilised to introduce 27 unique substitution mutations into the highly conserved fusion peptide loop region (amino acid positions 98–111) among DENV serotypes and flaviviruses, which successfully yielded seven stable mutant viruses. The majority of the stable mutant viruses (G102S, G104S, L107F, L107M, F108W) replicated less efficiently than the wild type virus despite exhibiting different growth kinetics in mosquito (C6/36) and mammalian (Vero and K562) cells, possibly indicating host-specific differences in fusion requirements. However, L107F and L107M mutations were observed to be unstable as they partially reverted to wild type phenotype in Vero cells and in both Vero and K562 cells, respectively. Additionally, introducing substitution/deletion mutations to these selected amino acid residues located within the fusion loop significantly reduced fusion efficiency. The mutations appeared to abrogate the fusion capacity of mutant viruses, which subsequently resulted in the production of non-infectious virus particles or lethal mutation phenotypes or viruses that displayed impaired replication efficiency in cell cultures [90]. Several other mutant viruses (W101F, L107A, F108A) demonstrated amplification of viral RNA in V-0 transfections, but intriguingly produced viruses that could not initiate a second round of infection (V-1), indicating that these mutants might be producing non-infectious viral particles. In the F108A mutant, upon entering the cells via receptor-mediated endocytosis, non-infectious mutant F108A particles were observed to be trapped in the endosomal compartment, failing to escape via fusion from the endosomes due to a defect in fusion activity. Thus, the F108A viral particles were likely being degraded in the lysosomes before degradation of the capsid to release the RNA genome to initiate replication. In summary, this comprehensive study highlighted the importance of the fusion peptide of DENV-2 in membrane fusion and viral replication.

The E protein of flaviviruses contains four main hinge regions (H1 to H4) that have been suggested to be essential for viral fusion, infection, and replication. To validate this, Butrapet and colleagues (2011) conducted mutagenesis studies by introducing selected substitution mutations to 15 amino acid residues across the four hinge regions and produced cDNA infectious clones [91]. A substitution mutation from alanine to glutamic acid residue at highly conserved amino acid residue 54 (A54E) among all flaviviruses led to a slight delay in viral replication and increased the fusion pH threshold. Three mutations (G266W, I270W, G281W) within the H4 hinge region were identified as molecular determinants for DENV-2 replication in both mosquito and mammalian cells. A mutant virus with a mutation at residue 280 (T280Y) displayed significantly lower replication efficiency than the wild type in C6/36 cells [91]. The glycine residues located at both amino acid positions 266 and 281 of the E protein are highly conserved among the DENV serotypes, whilst an isoleucine residue at position 270 is highly conserved among the DENV-1, DENV-2 and DENV-3 serotypes, as a valine is found in DENV-4. The threonine residue located at position 280 is found in DENV-2 only, whilst an alanine is present in the remaining three DENV serotypes. Lin et al. (2011) investigated the roles of the stem region (amino acid positions 398–446) of the E protein in viral assembly, replication, and entry [92]. Introducing proline mutations to several residues within the stem region severely impaired the formation of virus-like particles (VLPs) and affected prM-E heterodimerisation, which is essential for proper viral assembly. Four proline mutations (I398P, T405P, F429P, L436P), when individually incorporated into a full-length infectious clone, displayed a marked reduction in viral infectivity and spread when compared to the wild type. However, these mutations were deemed unstable as continuous passaging of these mutant viruses in Vero cells led to the rise of adaptive mutations that reestablished viral replication.

The introduction of two alanine substitution mutations (G296A, S298A) at two highly conserved residues within the DI-DIII polypeptide linker led to a significant severe impairment in viral assembly, completely abrogating the production of infectious virions [93]. Interestingly, viral entry remained unaffected despite these linker mutation modifications, or its interaction between the E and prM, highlighting that the linker is indispensable in viral assembly. Despite these mutations demonstrating impairment in viral assembly, they are not suitable mutations to be introduced to develop a new live attenuated vaccine candidate. Upon viral entry, these mutations within the linker were postulated by the authors to impede viral assembly, thus leading to the abolishment of viral particle production. If the severely attenuated mutant viruses were unable to infect their target cells to replicate in a vaccinated human host, they would eventually lose their ability to trigger an immune response to generate immunity against the virus. In non-ADE infection, following virus entry via clathrin-mediated endocytosis, it is essential that the DENV escapes the endosome and releases its nucleocapsid into the cytoplasm for viral replication. In the case of ADE-associated DENV infection, the binding of virus–antibody complexes to FcγR receptor might enable them to enter cells via phagocytosis [94]. Following cellular entry via the endosomal pathway, successful infection by virus–antibody complexes depends critically on viral membrane fusion within the endosomes. Chotiwan et al. (2014) employed a panel of DENV-2 E protein mutants to elucidate residues essential for viral entry during infection of FcγRIIA-expressing cells by virus–antibody complexes under ADE conditions [95]. The FcγRIIA is potentially involved in concentrating DENV–antibody complexes on cells. Two temperature-sensitive mutants carrying G104S and L135G mutations in the fusion loop and hinge region of the E protein, respectively, demonstrated that membrane fusion was indispensable for infectivity under both non-ADE and ADE circumstances. On the other hand, abrogating the fusion loop only led to a decrease in replication efficiency, whilst it was dispensable for both non-ADE and ADE infections.

A recent phylogenetic study conducted by Jiang et al. (2024) identified three non-synonymous mutations in three conserved residues (V324I, V351L, V380I) within the DENV-1 E-DIII domain that might be important for receptor binding during the cell entry process [96]. The identified mutations appeared to have emerged between the 1970s and 1990s and they were consistently inherited and expanded into contemporary strains during the 21st century. Utilising reverse genetics approaches, the reverse mutations (I324V, L351V, I380V) in the wild type DENV-1 strain replicated less efficiently, and displayed lower infectivity and weaker binding ability to host cells than the wild type virus in mammalian cells, indicating that these mutations could influence viral infectivity, binding capacity to target host cells and virion stability. Rani and colleagues (2024) recently evaluated the roles of highly conserved histidine residues located within the DENV E protein in terms of DENV maturation and secretion [97]. Substituting these histidine residues with alanine (H144A, H244A, H261A, H282A) led to a severe reduction in VLP formation by more than 50% compared to the wild type E, suggesting these specific histidine residues are critical for viral maturation and secretion. Taken together, these studies would provide new mutations to be validated in mice or monkeys via in vivo studies before being considered as mutation(s) to be introduced into a new live-attenuated dengue vaccine candidate.

## 11. Non-Structural 1 (NS1)

NS1 is a 48 kDa multifunctional non-structural glycoprotein that exists in two forms: an intracellular membrane-associated (mNS1) dimeric species localised in vesicular compartments or a secreted extracellular hexameric conformation (sNS1) [98,99]. The mNS1 is critically involved in viral replication by localising and engaging with components of the replication complexes in both insect and mammalian cells [100,101,102,103,104,105,106,107]. The sNS1 assembles into a hexameric lipoprotein particle, forming an open-barrel hexameric channel, with its hydrophobic core densely packed with lipid compositions similar to those of plasma-derived high-density lipoproteins (HDLs), which regulate vascular homeostasis [108,109]. Following the glycosylation of the NS1 monomer, the glycosylated NS1 monomer rapidly dimerises and is then targeted to three main sites: viral replication sites within the ER, plasma membrane, and the trans-Golgi network [98,110,111,112,113]. Cryo-EM studies revealed that the DENV NS1 dimer 3D structure is organised into three main domains: a hydrophobic N-terminal β-roll domain (amino acid positions 1–29), a central Wing domain (amino acid positions 38–151) flanked by two flexible linker subdomains (amino acid positions 30–37 and 152–180) and an extensive core β-ladder domain (amino acid positions 181–352), spanning the dimer length and are analogously arranged like rungs of a ladder [114,115].

NS1 protein has been identified to play important roles in viral RNA replication, due to its inherent participation in the formation of viral replication complexes [100,101]. Scaturro and colleagues (2015) utilised a DENV genome luciferase reporter construct to evaluate the impact of 46 NS1 point mutations within highly conserved residues on the DENV life cycle in terms of viral replication and infectious virus production [23]. Indeed, 23 of the NS1 selected mutations were predominantly located within the wing domain and β-ladder domain, and these mutations led to complete abrogation of viral replication activity. The 23 NS1 mutants also included five cysteine residues (C4A, C55A, C179A, C291A, C312A) that were shown to be involved in the formation of disulphide bonds and stabilising the NS1 fold. The alanine substitution mutations on the cysteine residues corroborated with a previous study by Fan et al. (2014), which demonstrated that the alanine substituted cysteine mutants (C4A, C55A, C291A) fully abrogated viral replication and growth, indicating these highly conserved cysteine residues might be essential in viral replication [116]. A few of the selected NS1 mutants (W8A, E142A, E219A, R314A) displayed a minor decrease in viral replication efficiency compared to the wild type. A group of alanine substitution mutations (S114A, W115A, D180A, T301A) led to a significant decrease in infectious virus production (up to ~2.5 log10 reduction compared to the wild type) but there were intriguingly minimal effects on viral replication, with a possible explanation being that impairment of infectious DENV production might be due to disruptions of NS1 interactions with DENV structural proteins. The interaction of NS1 with a newly discovered NS4-2K-4B cleavage intermediate was shown to be essential for viral RNA replication [117]. Introducing alanine substitution mutations to two residues (G161A, W168A) previously identified by Scaturro et al. (2015) completely abrogated viral replication activity and led to a near total loss of the interaction, strongly highlighting that the NS1-NS4-2K-4B association is indispensable for viral RNA replication [23,117].

The sNS1 has been heavily associated with DENV severe disease pathogenesis since its discovery, and it was detected at significantly elevated levels in the sera of DENV-infected patients [118,119]. A substitution mutation from a highly conserved threonine residue to serine at amino acid position 164 of the NS1 (T164S) displayed decreased production of infectious DENV in mammalian cells but comparable replication efficiency to the wild type virus [120]. The mutation appeared to enhance the secretion of sNS1 from infected cells, possibly suggesting that the mutation influenced NS1 secretion efficiency, rather than viral replication activity. Interestingly, infection of human PBMCs with the T164S mutant virus resulted in elevated production of proinflammatory cytokines when compared to the wild type virus and additionally led to more disease severity and rapid mortality in the immunocompromised AG129 mice model. Molecular dynamics simulation predicted that the substitution mutation at position 164 of the NS1 destabilised the secreted NS1 structure, potentially affecting its function and interactions, further indicating that this mutation might not be suitable to be included in a new live attenuated vaccine candidate. Utilising random point mutagenesis and split luciferase assay approaches, 10 NS1 point mutations were randomly identified to be essential for NS1 secretion, with follow-up in silico analysis revealing that they were predominantly located within the β-ladder domain of the NS1 [109]. Further studies investigating the mutational influences on the DENV replication cycle revealed that V220D and A248V DENV-2 NS1 mutants did not support viral replication and infectious virus production, indicating that they were unsuitable as potential candidates in the attenuation of DENV.

## 12. Non-Structural 2A (NS2A)

NS2A is a 22 kDa highly hydrophobic transmembrane protein comprising five integral transmembrane segments (pTMS3, pTMS4, pTMS6, pTMS7, pTMS8) that stretch throughout the lipid bilayer of the ER membrane [121]. NS2A has been widely reported to be important for viral assembly, pathogenesis, membrane remodelling, viral replication, and antagonism of the host immune response [122]. Following DENV polyprotein translation, the termini of NS2A undergo distinctly different processing. Located within the ER lumen, the N-terminus is proteolytically cleaved by an undefined membrane-bound host protease, whilst the C-terminus is cleaved in the cytoplasm by the viral NS2B3 protease, giving rise to the mature DENV NS2A [123]. During virion morphogenesis, the DENV NS2A binds to the 3′ UTR and proceeds to recruit nascent viral RNA from the replication complex to the site of virion assembly [124]. At this site, the cleavage of the C-prM protein precursor promotes the packaging of the viral genome with capsid proteins. Concurrently, the prM and E proteins are incorporated into the ER membrane, leading to the budding and release of progeny virions. Interaction of NS2A with 3′ UTR leads to the recruitment of nascent viral RNA from the replication complex to the virion assembly site. Cleavage of C-prM promotes genomic RNA encapsidation with the capsid and facilitates the budding of the prM and E proteins into the ER, giving rise to progeny virions. The interaction between the NS2A and 3′ UTR facilitates localisation of the C-prM-E polyprotein and the NS2B-NS3 protease to the virion assembly site. Subsequent proteolytic cleavage activity by NS2B-NS3 and the host signalase process the C-prM-E polyprotein, yielding the mature C, prM, and E structural proteins.

To assess the biological role of the helix breaker within pTMS3, the arginine and proline residues at positions 84 and 85 of the helix breaker region, respectively, were substituted with mutations [121]. The arginine at position 84 was substituted by three different amino acid residues, thereby generating R84A, R84E, and R84S mutations, while the proline was substituted with alanine (P85A). R84A mutation did not abrogate viral RNA synthesis but blocked the intracellular formation of infectious virions, indicating a likely role in virion assembly. On the other hand, the R84E mutation appeared to attenuate both viral RNA synthesis and infectious virus production, whilst R84S did not affect viral RNA synthesis but led to a reduction in infectious virus production. P85A mutation which eliminated the helix breaker function, intriguingly did not influence viral replication or infectious virus production activity, highlighting that the helix breaker is dispensable for these roles in the DENV replication cycle. The arginine residue at position 84 is semi-conserved among DENV-1 and DENV-2 serotypes, whilst glutamine and proline residues are found at this position in DENV-3 and DENV-4, respectively. Conversely, the proline at position 85 is highly conserved among DENV serotypes except in DENV-4, where a glycine residue is found at this position. Mutagenesis of several highly conserved residues among flaviviruses identified two different sets of NS2A mutations, one set (G11A, E20A, E100A, Q187A, K188A) that impaired virion assembly but did not significantly affect RNA synthesis whilst the other set of mutants (D125A, G200A) specifically abolished viral RNA replication activity only [125]. Another set of NS2A mutations among highly conserved residues in NS2A (R24A, R26A, G46A, D52A, G69A, F81A, T97A, K99A, K135A) resulted in slight or no effects on viral replication activity, thus, they were not subsequently analysed.

Wu and colleagues (2015) introduced triple alanine substitutions in the C-terminal half portion of DENV-2 NS2A (pTMS4 to pTMS8) (amino acid positions 109–209) to identify key residues that are essential for the DENV life cycle in terms of viral assembly, RNA replication, and NS2A secretion [126]. Fascinatingly, these triple alanine mutants [CM20 (amino acid positions 163 to 165), CM25 (amino acid positions 178 to 180), CM27 (amino acid positions 184 to 186)] within the pTMS7 displayed >100-fold reduction in virus yield, smaller plaque sizes, distinctive defects in viral assembly and secretion compared to the wild type and exhibited mild decrease in viral replication, implying that pTMS7 plays an important role in the later stages of the DENV life cycle. The pTMS7 segment has been strongly suggested to interact with other transmembrane segments (pTMS4, pTMS6, pTMS8) to regulate viral replication activity. Approximately 43% of the NS2A mutants with mutations within the C-terminal half of NS2A exhibited replication efficiency like the wild type, indicating that these mutations did not influence viral replication activity. However, continuous passaging of NS2A mutant-infected cells led to a 1000-fold increase in virus titers compared to the original NS2A mutant, which, upon sequencing, led to the discovery of a compensatory mutation at amino acid position 181 of NS2A (L181F). Wu et al. (2017) identified amino acid residues within the N-terminal half of DENV-2 NS2A (pTMS1 to pTMS4) that were crucial for the roles of NS2A in the DENV life cycle [127]. Indeed, five triple alanine substituted NS2A mutants [NM5 (amino acid positions 16 to 18), NM7 (amino acid positions 21 to 23), NM9 (amino acid positions 27 to 29), NM17 (amino acid positions 50 to 52), NM19 (amino acid positions 56 to 58)] exhibited >1000 fold reduction in virus yield with no visible viral plaques formed but exhibited wild type-like replication and infectious virus production compared to the wild type. Additionally, these NS2A mutants also resulted in notable defects in virus-induced cytopathic effects (CPE). A compensatory mutation in the NS2A L181F was surprisingly identified to rescue viral infectivity and infectious virus production in CPE-defective and lethal-mutant viruses [126,127]. Thus, the identification of residues essential in the role of NS2A in the DENV life cycle could help to facilitate the design of novel live-attenuated vaccine candidates.

## 13. Non-Structural 2B (NS2B)

NS2B is a 14 kDa (130 amino acids long) membrane-bound protein that serves as an important cofactor for the NS3 protease [128]. NS2B contains four transmembrane helices (α1–α4), and a non-helical, highly conserved region located between the α2 and α3 helices that is essential for its cofactor function in NS3 protease activity [129]. Additionally, this conserved domain is proposed to interact with NS1 via β-strand formation, facilitating the assembly of the NS2B–NS3 complex, which is essential for the DENV polyprotein processing into non-structural proteins (NS2B, NS3, NS4A, NS4B, NS5). Being a component of the DENV NS2B3 protease complex, the NS2B has been implicated in targeting the cytosolic DNA sensor cyclic GMP-AMP synthase (cGAS) for lysosomal degradation [130]. This process subsequently led to the disruption of cGAS–STING signalling pathway, which in turn inhibited type I interferon (IFN) production and promoted viral replication.

In a study to identify regions within the NS2B that were important for the NS2B–NS3 complex-mediated protease activity, a 40-amino acid long domain within the DENV-4 NS2B (DENV-4 amino acid positions 1396 to 1435) was identified to be essential for NS2B protease activity [131]. This hydrophilic domain appears to be highly conserved among the four DENV serotypes, suggesting a common polyprotein processing mechanism among the flaviviruses. Despite being important in viral replication and acting as a cofactor for NS3 protease activity, there is a lack of mutagenesis studies of the DENV NS2B to elucidate its functional domains, structural stability, and other undefined roles in the DENV life cycle. While it is understood that there may be underlying challenges in manipulating the NS2B without impacting its structural integrity and disrupting viral viability, additional mutagenesis studies could provide future insights into its comprehensive roles in the DENV life cycle that could be effectively targeted for live-attenuated vaccine development.

## 14. Non-Structural 3 (NS3)

NS3 is a 72 kDa large multifunctional protein (618 amino acids long) that has been extensively studied [85]. It is the most highly conserved protein among DENV with approximately 77% sequence identity among the four DENV serotypes, making it a promising target for broad-spectrum vaccine development against DENV [132]. The N-terminal serine protease domain (amino acid positions 1–168), together with its cofactor NS2B, is responsible for the proteolytic activity of NS3 through the formation of an NS2B–NS3 protease complex. The C-terminal domain of NS3 (amino acid positions 180–618) exhibits three distinct enzymatic activities through its nucleoside 5-′triphosphatase (NTPase), RNA helicase, and RNA 5′-triphosphatase (RTPase) domains [133]. The NS3 helicase domain utilises ATP hydrolysis to unwind double-stranded RNA replication intermediates. This activity is powered by the NTPase domain, which hydrolyses nucleoside triphosphates to generate the required energy. The NS3 RTPase activity plays a critical role in processing 5′-triphosphorylated viral RNA by cleaving its γ-β phosphoanhydride bond, a prerequisite for 5′ UTR viral RNA capping, genome stability, and viral replication [85]. Thus, these three subdomains function collaboratively to facilitate genome replication and viral RNA synthesis, highlighting the pivotal role of the C-terminal domain in the DENV life cycle.

A DENV-2 NS3 mutagenesis study introduced 46 amino acid substitutions within the NS3, with particular focus on the NS3 Box 3 and 4 regions (amino acid positions 126–184) [134]. Of the 46 mutations,12 (T134D, S135A, S135C, G148A, L149A, L149R, Y150A, Y150V, Y150H, G151A, G153A, G153V) were able to inhibit protease activity, nine (D129K, D129R, D129I, F130A, F130S, G133A, G136A, N152A, N152Q) of them significantly reduced protease activity, 14 (V126A, D129E, D129S, D129A, F130Y, T134A, I139L, I139A, I140A, G144P, L149I, Y150F, V154A, V155A) demonstrated decrease in cleavage efficiency and 11 showed wild type levels of activity. Substitutions at the catalytic serine [(S135A) and other identified ultra-conserved residues (G133, G136, G148, L149, G153)] among flaviviruses within the NS3 completely abrogated NS3 protease activity, illustrating their importance in regulating protease function. Thus, mutations that were identified to significantly reduce protease activity could be potentially targeted in the attenuation of DENV. By introducing them into a full-length infectious cDNA clone, these mutations could be comprehensively studied regarding their mutational effects on viral replication and viral pathogenicity. Nearly all the NS3 residues that were substituted led to a significant or slight decrease in cleavage activity, and they were highly conserved among the DENV serotypes and flaviviruses, except for the V126 and V140 residues. Both V126 and V140 residues are semi and highly conserved among the DENV serotypes, respectively. In a related study, Matusan and colleagues (2001) investigated the effects of several mutations within DENV-2 NS3 on protease activity and viral replication [135]. Substitution from highly conserved methionine to phenylalanine at amino acid position 283 of motif II of the NS3 (M283F) intriguingly displayed reduced ATPase activity but increased helicase activity, suggesting that both enzymatic activities were functionally uncoupled by the mutation within motifs. Additionally, M283F mutant viruses demonstrated a temperature-sensitive phenotype at higher temperatures, which led to a reduction in RNA synthesis, viral protein synthesis, and viral yield. Thus, the M283F mutation could be useful to further investigate its role in viral replication and developing new live-attenuated vaccine candidates against dengue.

DENV-4 mutant viruses harbouring a substitution mutation from an aspartic acid residue to asparagine at amino acid position 192 (D192N) within the DENV-4 NS3 exhibited distinctively small plaques, temperature-sensitive phenotype, and attenuation in mammalian cells and animal models [136]. The D192N mutant virus showed significant viral attenuation in the brains of 6–7-day-old suckling mice (>10,000-fold reduction compared to the wild type) and in 6-week-old severe combined immunodeficiency disease (SCID) mice transplanted with Huh-7.5 cells that were inoculated with 10^4^ PFU of DENV-4 virus. There was approximately ~3.6 log10 PFU/mL reduction in viremia when compared to the wild type DENV-4. Thus, it was proposed that the NS3 D192N mutation is a key attenuating mutation within a highly conserved residue among DENV serotypes and could provide an avenue for developing a novel tetravalent live-attenuated DENV vaccine. In NS3, both the N-terminal serine protease domain and the C-terminal RNA helicase domain are connected by an 11-amino acid linker (ERIGEPDYEVD) [137]. Utilising a DENV-2 replicon system, substitution mutation from a proline to glycine residue at amino acid position 176 (P176G) of DENV-4 NS3 or P174G in DENV-2 NS3 which led to a 70% reduction in luciferase reporter activity and decreased viral RNA synthesis when compared with the wild type replicon, highlighting that the NS3 linker flexibility was indispensable for polyprotein processing and DENV replication cycle. The findings provided important implications in the development of a live-attenuated vaccine that specifically targets the NS3 protein. The proline residue at amino acid position 176 (DENV-4) or position 174 (DENV-1) is highly conserved among DENV serotypes. Tay and colleagues (2015) also identified a residue within DENV NS3 that is highly conserved among DENV serotypes and other flaviviruses [132]. Asn-570 was found to be essential for its interaction with NS5 in the DENV life cycle. Substitution of the asparagine residue with alanine at amino acid position 570 (N570A) of the NS3 in DENV-2 cDNA clone was shown to decrease viral protein production, reduce viral RNA replication, significantly reduce positive-strand RNA synthesis, and led to severely abrogated infectious virus production. Interestingly, it did not completely abolish negative-strand RNA synthesis, suggesting that NS3–NS5 interaction is crucial for the replication efficiency of the viral genome, especially in the synthesis of positive-strand RNA from the negative-strand template. Overall, these findings have identified potential new targets for the development of novel live-attenuated vaccine candidates.

Gebhard et al. (2016) conducted a comprehensive mutagenesis study to dissect the roles of DENV-2 NS3 in infectious virus production and viral replication [138]. Within a DENV luciferase reporter system, single, double, or triple alanine mutations were specifically introduced into various domains of NS3 that have been suggested to be important for the DENV life cycle. A substitution from a highly conserved tryptophan residue among DENV serotypes to an alanine residue at position 344 of NS3 (W344A) resulted in severe reduction of viral replication efficiency and complete abrogation of infectious virus production. Intriguingly, a triple alanine mutation (E574A/E575A/N576A) at highly conserved residues at positions 546–576 caused a slight delay in viral replication before exhibiting replication efficiency that was comparable to the wild type. However, this triple alanine mutation notably decreased the production of infectious viral particles (>10,000-fold lower than the wild type). Two double alanine mutations (P10A/P12A, K63A/R64A) abolished infectious virus production without affecting viral replication activity. These findings demonstrated that molecular determinants of infectious DENV production were located separately in DENV NS3, possibly illustrating that NS3 serves as a platform for viral and host interactions and is associated with infectious DENV production. The proline residue at amino acid position 10 is ultra-conserved among flaviviruses, whilst proline at amino acid position 12 is not conserved among DENV serotypes. The lysine residue at position 63 is also highly conserved among DENV-1, -2, and -3 serotypes, as glycine is found only in the DENV-4 serotype, whilst the arginine at position 64 is ultra-conserved among flaviviruses except in Japanese encephalitis virus (JEV). Another study identified several residues within the DENV NS3 helicase to be essential for RNA base recognition for RNA–protein interactions during flavivirus replication [139]. DENV NS3 structural studies revealed that both D290 and R538 residues specifically interacted with RNA G2 and G5 bases, respectively, and these interactions are important for optimal helicase activity for viral replication. Indeed, mutating these residues with alanine residues significantly attenuated viral replication and infection as observed through the formation of smaller plaques/undetectable plaques and reduction in RNA synthesis. The aspartic acid residue at position 290 (D290) of the NS3 is ultra-conserved among flaviviruses, whilst the arginine residue at position 538 (R538) is highly conserved among DENV-1, -2, and -3 serotypes, as a lysine residue is found at this position in the DENV-4 serotype. In summary, this study demonstrated the importance of specific RNA–protein interactions in DENV replication, and these new findings could be capitalised on for the design of novel live-attenuated dengue vaccines.

## 15. Non-Structural 4A (NS4A)

NS4A is a 16 kDa hydrophobic transmembrane protein (125 amino acids long) that is localised on the ER membrane [140]. The NS4A protein is composed of an N-terminal cytoplasmic region (amino acid positions 1–48), three membrane-associated regions (amino acid positions 50–73, amino acid positions 76–89, amino acid positions 101–127), a small loop that exposes the NS4A-2K cleavage site (amino acid positions 123–130) and a C-terminal fragment known as 2K that functions as a signal peptide sequence for NS4B translocation into the ER lumen [140,141]. Following the translocation of NS4B, the 2K fragment is cleaved from the N-terminal of NS4B by the NS2B-3 protease present on the ER membrane cytosolic side, whilst the C-terminal domain of NS4B is formed by the host signalase within the ER lumen. The proteolytic cleavage of the 2K fragment is involved in the modification of the cytoplasmic membrane, leading to virus-induced structure formation, which is likely to harbor the viral replication complex. pTMD1 (amino acid positions 51–73) and pTMD3 (amino acid positions 105–120) span through the ER membrane, whilst pTMD2 (amino acid positions 80–96) is embedded in the lumen of the ER membrane [140]. These membrane domains form a structure where both NS4A N- and C-termini are oriented towards the cytoplasm, where cleavage by DENV NS2B-3 protease occurs [85]. The NS4A C-terminal domain encodes a signal peptide (the 2K fragment or pTMD4) which enables the translocation of adjacent NS4B into the ER lumen. The N-terminal region was shown to interact with the ER membrane and might be involved in NS4A-induced membrane curvature, thus contributing to the formation of the membranous DENV replication complex [142].

Secondary structure analysis identified an N-terminal amphipathic α-helix (AH) spanning the first 48 amino acids of NS4A that is likely to be essential for NS4A oligomerisation and viral replication [143]. The AH is important for the ability of NS4A to bind to membranes and form oligomers, which are required for the formation of viral replication complexes. The introduction of mutations to disrupt the AH severely impaired viral replication and membrane binding, indicating the importance of this α-helix in the DENV life cycle. Co-immunoprecipitation (co-IP) experimental assays successfully mapped amino acid residues located at positions 40–76 of NS4A and the amino acid at positions 84–146 of NS4B to be the molecular determinants of the NS4A–NS4B interaction, which was essential for DENV replication [144]. Substituting 11 highly conserved NS4A residues (amino acid positions 40–76) among DENV serotypes with alanine/glycine residues identified specific residues that were deemed to be important for NS4A–NS4B interaction and DENV replication. Several mutations (E53A, L57A, G67A) led to a decrease in viral yields and smaller plaques when compared to the wild type, except for E53A, which formed plaques that could only be detected by immunostaining. On the other hand, both A40G and A44G mutant viruses displayed a severe reduction in viral replication (<1000 PFU/mL virus), with A40G forming smaller plaques than the wild type virus, whilst the A44G interestingly displayed plaque sizes that were comparable to the wild type. Lastly, several mutations (T54A, L60A) almost or completely abolished viral replication with no IFA-positive cells, and undetectable plaques, and significantly affected the NS4A–NS4B interaction. Thus, the study concluded that mutations that could impede NS4A–NS4B interaction would severely abolish viral replication, highlighting the influence of this interaction on the DENV replication cycle. Targeting the NS4A–NS4B interaction could be favourably considered as a potential strategy for attenuating DENVs.

Co-IP experiments revealed the first transmembrane domain (TMD1, amino acid positions 50–76 as an important molecular determinant for NS4A oligomerisation [145]. Lee and colleagues (2015) conducted a comprehensive alanine scanning mutagenesis on 15 conserved residues spanning throughout DENV NS4A to evaluate their impacts on DENV replication. Six mutations (P14A, K20A, D26A, P49A, K80A, E122A) completely abolished viral replication whilst three mutations (R12A, P121A, E124A) exhibited severely attenuated viral replication. Both E50A and G67A mutations appeared to moderately reduce viral replication and reduce NS4A oligomerisation, leading to a decrease in the stability of NS4A mutant proteins. The mutant viruses were able to revert to the wild type following continuous passaging. The E50 residue is highly conserved in DENV-2, -3, and -4 serotypes, as D50 is only found in the DENV-1 serotype, whilst the G67 is ultra-conserved among DENV serotypes and flaviviruses. The Wing domain of NS1 was identified to interact with a short fragment of NS4A (amino acid positions 35–61), which includes highly conserved tyrosine and histidine residues at amino acid positions 41 and 43 of NS4A, respectively [146]. Substituting the tyrosine residue with alanine (Y41A) rendered the Y41A mutant non-viable, with no viral replication being detected, whilst substituting with phenylalanine (Y41F) severely attenuated the virus, as slower replication kinetics and smaller plaques were observed in comparison with the wild type. Conversely, alanine substitution of the histidine at position 43 (H43A) was able to resemble the wild type phenotype, given that this specific residue has been established to be important for NS1–NS4A interaction in Yellow Fever Virus (YFV). A combination of biochemical, imaging, and genetic approaches was comprehensively utilised to identify the molecular determinants of NS4A that were associated with DENV replication [147]. The majority of the mutations introduced into conserved residues in NS4A led to severe impairment or complete abrogation of viral replication. These replication-defective mutations also disrupted the formation of vesicle packets (VP) and interactions between NS4A-2K-4B and NS3, which is a major component of the viral replication complex. In summary, this study highlighted the importance of specific NS4A residues in DENV replication, VP formation, and protein interactions, which could be greatly considered to be introduced into new live-attenuated dengue vaccine candidates.

## 16. Non-Structural 4B (NS4B)

NS4B is a 27 kDa hydrophobic transmembrane protein (248 amino acids long) that is involved in viral replication, membrane remodelling of the ER, and immune evasion [148]. NS4B is highly conserved across all four DENV serotypes. NS4B has 11 helices, in which only five transmembrane (TM) helices are predominantly involved in membrane rearrangements and hence are important for the DENV life cycle [85]. Based on the established NS4B membrane topology model devised by Miller and colleagues (2006), the NS4B constitutes three regions, an N-terminal domain (amino acid positions 1–40) that resides in the ER lumen, a central hydrophobic region (amino acid positions 100–160) that anchors the NS4B in the ER membrane and a C-terminal region (amino acid positions 200–248) that is located in the cytoplasm [148]. The NS4B is known to interact with other viral proteins, mainly the NS3 and NS4A, in forming the viral replication complex.

A study investigated the interaction between NS3 and NS4B by identifying the molecular determinants of this interaction and the role of NS4B in viral replication [149]. Surface plasmon resonance (SPR) assays identified the major determinants of this NS3–NS4B interaction to be within the subdomains 2 and 3 of NS3 helicase and the cytoplasmic loop of NS4B. Further nuclear magnetic resonance (NMR) studies revealed 12 amino acids within the NS4B cytoplasmic loop (amino acid positions 129–165) that participated in the interaction with NS3, in which five of the 12 residues (G124, Q134, G140, N144, D154) are ultra-conserved among flaviviruses. Alanine substitution mutation of the glutamine residue (Q134A) was lethal, as no viral replication was observed, but the mutant rapidly reverted to the wild type upon continuous passaging. G140A mutation significantly impaired viral replication, but further sequencing revealed that the mutant virus reverted to the wild type, suggesting strong selective pressure to maintain the glycine residue at position 140. Alanine substitution mutation at position 144 (N144A) led to a 1000-fold reduction in virus titers and exhibited plaques slightly smaller than the wild type. Both G124A and D154A mutations appeared to have no impact on viral replication but exhibited slightly smaller plaques than the wild type, like N144A. Thus, these residues within the NS4B cytoplasmic loop, particularly Q134 and G140, are likely to be essential for DENV replication. NS4B was identified to be an N-glycosylated protein with two N-glycosylation sites at amino acid residues 58 and 62 [150]. To identify the biological roles of these NS4B N-glycosylated residues, single and double substitution mutations (N58Q, N62Q, N58Q/N62Q) were introduced into these ultra-conserved asparagine residues among DENV serotypes before incorporating them into full-length DENV-2 infectious clones. N58Q single mutation led to a mild reduction in viral replication, whilst N62Q displayed a significant reduction in viral replication. On the other hand, the double N-glycosylated mutations resulted in a severe reduction in viral replication and infectious virus production efficiency compared to the wild type, but the impaired replication activity could be rescued via trans-complementation by compensatory mutations such as S59Y, S59F, T66A, and A137T that compensated for structural defects. These findings highlighted the importance of mutations in NS4B N-glycosylation in the DENV life cycle and might have the potential to be included in a live-attenuated vaccine strain.

Chatel-Chaix et al. (2015) utilised a combination of proteomic and genetic approaches to elucidate the role of critical residues within the NS4B that were crucial for its NS4B–NS3 interaction in the DENV life cycle [151]. Alanine substitution mutations on 33 conserved NS4B residues were introduced before individually incorporating the mutations in full-length infectious DENV genomes. The majority of the substitution mutations, including the N-glycosylation N62A mutation, were shown to be detrimental to viral replication. Intriguingly, six mutants (I18A, P23A, N58A, K135A, P157A, P159A) demonstrated robust RNA replication compared to the wild type, whilst three mutants (N144A, V147A, G149A) were severely attenuated, suggesting that these residues were indispensable for the role of NS4B in the DENV life cycle. The N144, V147, and G149 residues within the NS4B are ultra-conserved among flaviviruses. Bui et al. (2018) reported the identification of different amino acid residues at position 116 (116A, 116M, 116V) of NS4B in clinical DENV-1 isolates derived from dengue-infected patient sera in Vietnam [152]. Intriguingly, this newly identified viral determinant at position 116 demonstrated varying effects on DENV replication and viral fitness in mammalian and mosquito cell lines. DENV-1 NS4B-116A and NS4B-116M mutant viruses demonstrated increased virus growth in mammalian cells but were observed to display significantly reduced viral replication in mosquito cells, in contrast to what was observed for the NS4B-116V mutation. Thus, this study highlighted the importance of the amino acid residue at position 116 in NS4B in DENV replication and DENV differential inter-host adaptation, offering another potential candidate for the development of live attenuated vaccines. Serial passaging of a DENV-2 strain in mammalian cells led to the identification of several adaptation mutations throughout DENV non-structural proteins, with an NS4B G124A mutation among those identified [153]. Viruses harbouring the NS4B G124A mutation exhibited enhanced viral growth and increased viral titers in mammalian cells compared to the wild type, but no plaques were formed in C6/36 mosquito cells, suggesting that this mutation had host-specific effects on DENV proliferation and viral genome replication.

## 17. Non-Structural 5 (NS5)

Located within the C-terminal of the DENV polyprotein, the NS5 is the largest protein (900 amino acids long; 105 kDa) that is encoded by the flavivirus genome and is the most highly conserved flaviviral protein [154]. NS5 constitutes two domains, the C-terminal domain and the N-terminal domain, interconnected by a flexible linker that enables them to function cooperatively or independently [155]. NS5 is a multifaceted protein with two main domains. An N-terminal methyltransferase (MTase) domain that is responsible for methylation of viral RNA for host immune evasion and possesses three enzymatic activities, guanine N7 methyltransferase, guanylyl-transferase, and ribose 2′O-methytransferase activities that are prerequisite for viral RNA 5′ capping [156,157]. The C-terminal domain of NS1 contains motifs characteristic of the RNA-dependent RNA polymerase (RdRp) that is required for the de novo synthesis of viral RNA by utilising positive-sense (+) viral RNA as a template [156,158]. The RdRp activity of NS5 mediates viral RNA synthesis through three distinct stages: de novo initiation, transition and elongation [158]. Being a key component of the human innate antiviral response, the type I interferon (IFN) response activates effector cells through STAT2 and is mediated via STAT2 signalling, leading to suppression of viral replication [159]. However, DENV NS5 counteracts this essential pathway by binding to STAT2 protein and inducing its proteolytic degradation.

Both guanine N7 methyltransferase (N7 MTase) and ribose 2′O-methyltransferase (2′-O) enzymatic activities were mediated by a single S-adenosyl-L-methionine (AdoMet) binding site [160]. A site-directed mutagenesis approach was employed to introduce point mutations within the NS5 N7 MTase domain, focusing on residues essential for AdoMet binding or those potentially involved in NS5 structural conformation. Mutations targeting AdoMet binding-associated residues (S56A, W87I, W87K, D131A) severely reduced or completely abolished both N7 and 2′O MTase activities, indicating that one or both MTase activities were essential for viral replication. Several mutations targeting residues outside of the AdoMet binding pocket (K46A, R47A, E49A, G48A, G48P) displayed N7 MTase activity comparable to the wild type N7 MTase, whilst completely abolishing 2′-O MTase activity. Most importantly, these mutations appeared to be lethal for viral replication, possibly suggesting that these residues were essential for conformation and stability of the MTase domain. Both mutagenesis and biochemical studies were conducted to determine the role of specific amino acid residues in the RdRp domain of DENV NS5 in RNA synthesis and viral replication [161]. Mutagenesis analysis of several basic residues substituted with alanine led to the identification of several NS5 mutants (R325A, R519A/K523A, R769A, K840A/R841A) with high RNA synthesis activity in mammalian cells, but they exhibited delayed or impaired replication activity when incorporated into DENV2 infectious clones. Another double mutant (R361A/K370A) demonstrated reduced RNA polymerase activity and delayed viral replication, which could be thoroughly explained by a probable defect in the enzymatic function and localisation of the NS5 protein.

The DENV-1 to DENV-4 NS5 proteins were revealed to localise differently in DENV-infected cells, with the DENV-1 NS5 protein predominantly in the cytoplasm while DENV-2, -3, and -4 NS5 were localised in the nucleus [162,163]. Gene swapping approach in tandem with confocal light scanning microscopy analysis unexpectedly identified the NS5 C-terminal 18 residues at positions 883–900 (Cter_18_) to be minimally sufficient to enable nuclear localisation of NS5 [163]. Mutagenesis studies to identify the molecular determinants within Cter_18_ that were essential for NS5 subcellular localisation were performed by introducing alanine substitutions into DENV-2 cDNA clones. Double alanine mutations (K887A/R888A, R890A/R891A) when introduced into DENV-2 NS5, exhibited cytoplasmic localisation and severely impaired viral replication, with the K887A/R888A double mutations being lethal. Substitution of the arginine at position 888 with either alanine (R888A) or glutamic acid (R888E) residue resulted in the production of non-viable mutant viruses, while a lysine substitution (R888K) led to a severely attenuated phenotype, with reduced viral replication, but NS5 remained localised to the nucleus. In summary, the NS5 Cter_18_ is involved in regulating viral replication and subcellular localisation, with the role of the ultra-conserved residue R888 being indispensable for DENV replication. The highly conserved valine or isoleucine residue within the DENV NS5 interdomain linker (amino acid positions 263–272) has been hypothesised to regulate viral replication and maintain structural integrity [164]. Within a DENV2 infectious cDNA clone, DENV-3 NS5 V264G or DENV-2 NS5 (I265G) mutant virus demonstrated slight attenuation and reduced viral replication efficiency. However, substituting a proline residue instead of glycine at position 264 of DENV-3 NS5 (V246P) or DENV-2 NS5 (I265P) led to severe attenuation of the mutant viruses compared to the wild type. Insertion of a glycine (VGN) or proline (VPN) after V264 in DENV-3 NS5 led to the generation of mildly attenuated mutant viruses with enhanced RNA polymerase activity than the wild type, highlighting the role of the valine/isoleucine residue within the NS5 linker in regulating flexibility and conformational plasticity of NS5 required for viral replication. Thus, the NS5 linker region could be considered a prospective target for live-attenuated dengue vaccine development. The valine residue at position 264 of DENV NS5 is almost ultra-conserved among flaviviruses except in DENV-2, where an isoleucine residue is found at this position.

## 18. 3′ Untranslated Region (3′ UTR)

The 3′ untranslated region (3′ UTR) of DENV is approximately 400–600 nucleotides long and contains highly conserved structures [66]. The DENV 3′ UTR is important for viral pathogenesis, translation, and viral replication. The 3′ UTR can be divided into three domains; domain 1 comprises variable nucleotide sequences containing two nearly identical stem-loop structures (SLI and SLII); domain 2 is partially conserved, featuring two structurally similar dumbbell elements (DB1 and DB2) that contain both repeated conserved sequence 2 (RCS2) and CS2 elements; domain 3 is highly conserved across flaviviruses, characterised by two RNA elements: a small hairpin (sHP) and a terminal 3′ stem-loop (3′ SL) structure found in all flaviviruses [165,166,167,168]. Both SL and DB structures contain secondary structures, including pseudoknot (PK) interactions that delay genome degradation by nuclease activity [169]. The 3′ SL structure is known to interact with host and viral proteins to regulate viral RNA synthesis and translation [170]. During DENV infections, SLI and SLII are mainly involved in the production and accumulation of products of incomplete degradation of RNA by the host 5′-3′ exoribonuclease XRN1 activity and are known as subgenomic flavivirus RNAs (sfRNAs) [66,171]. These sfRNAs appeared to be important in counteracting host antiviral responses in mammalian and mosquito cells [172].

Both DENV-2 Thai and DENV-2 Nicaraguan strains were evaluated based on their rates of viral binding, entry, RNA stability, translation efficiency, and their ability to infect cells [173]. Low-passage Nicaraguan strains appeared to infect cells less efficiently and translate viral proteins, which led to the identification of specific differences in the 3′ UTR. Sequencing analysis identified 10 nucleotide changes within the 3′ UTR region, highlighting the importance of the 3′ UTR in viral translation efficiency. As such, these 10 nucleotide changes identified could be utilised to attenuate the wild type DENV strains. Wei and colleagues (2009) utilised the virus-induced reporter gene (VIRG) system and deoxyribozymes (DRzs) assays to target specific RNA sequences and secondary structures within the 3′ UTR of the DENV-2 genome and evaluated their roles on viral translation efficiency [174]. Deletion of the entire DENV-2 3′ UTR region (v-Δ3′ UTR) led to no detectable viral translation, indicating that the 3′ UTR is important for viral translation efficiency. Nucleotide segments from 10,663–10,677 nt to 10,709–10,723 nt within the DENV-2 stem-loop element were identified to be important for translation suppression, given that mutating these segments resulted in enhanced translation efficiency. These findings provided some insights into the mechanisms of DENV-2 translation regulation, which could be useful for live-attenuated dengue vaccine development.

The two DB structures within the 3′ UTR core region can form pseudoknot structures between identical five-nucleotide terminal loops, TL1 and TL2, and pseudoknot motifs PK2 and PK1, respectively, which are essential for modulating viral RNA function [175]. Conserved five-nucleotide deletions were introduced into TL1 (Δ5TL1) or TL2 (Δ5TL2) or both TL1 + TL2 (Δ5TL1Δ5TL2), and these led to a 70–80% decrease in replication in TL1, a 90% decrease in TL2, and severe attenuation for TL1 + TL2. In terms of RNA translation, Δ5TL1 did not influence translation, Δ5TL2 modestly affected translation, but Δ5T1Δ5T2 demonstrated a 60% decrease in translation, suggesting a cooperative synergy between TL1 and TL2. Additionally, introducing mutations in the conserved sequence 2 (CS2) within the 3′-DB resulted in reduced viral translation and replication, indicating that CS2 was important for both viral functions. It was proposed that these elements might interact with host factors to enhance viral RNA translation and replication. Filomatori et al. (2017) demonstrated that adapting DENV-2 in human or mosquito cells resulted in the production and accumulation of different species of sfRNAs regulated by specific RNA structures in the 3′ UTR, mainly the xrRNA1 and xrRNA2 structures [176]. Long sfRNA1 was predominantly produced in human cells, whilst shorter sfRNA3 and sfRNA4 were produced in virus-adapted mosquito cells. Specific mutations within the xrRNA2 structure led to the production of shorter sfRNAs (sfRNA3 and sfRNA4) that reduced viral fitness in human cells and could be outcompeted by long sfRNA1-producing viruses. As sfRNA1 was demonstrated to be essential for viral fitness in human cells, introducing mutations that would decrease sfRNA1 production could be desirable to produce attenuation of DENVs.

**Table 1:** Nucleotide changes in the DENV genome and the impact of these mutations in in vitro studies.

Genome Region	Position in the Genome	Mutation(s)	Consequences of the Mutation	References
5′ UTR	Nucleotide Δ82–87 deletions in DENV-4	Deletion of 6 nucleotides	Viable viruses with reduced RNA translation efficiency Generated small plaques in LLC-MK2 cells Failed to produce plaques in C6/36 cells	[68]
Capsid (C)	Amino acid position 204 in DENV-2	A204G	Retained defect in viral replication despite being rescued by compensatory activity	[74]
Capsid (C)	Double alanine mutations at amino acid positions 6 and 7	K6A/K7A	Reduced replication efficiency at approximately 5–50 times lower than wild type in PS cells Displayed reduced nuclear localisation in PS cells	[76]
Capsid (C)	Double alanine mutations at amino acid positions 73 and 74	K73A/K74A	Reduced replication efficiency at approximately 5–50 times lower than wild type in PS cells Total elimination of nuclear localisation in PS cells	[76]
prM/M	Amino acid position 39 in DENV-2	H39N H39R	Moderate decrease in viral replication efficiency (~2log10 lower than the wild type	[82]
prM/M	Amino acid positions 124, 128, 129 in DENV-1	E124A L128A R129A	Reduced assembly of VLPs and replicon particles >2-log reduction in amounts of replicon RNA	[83]
Envelope (E)	Amino acid positions 102, 104, 108 in DENV-2	G102S G104S F108W	Reduced growth in both K562 and Vero cells by 2 to 4.6logs at 37 °C	[90]
Envelope (E)	Amino acid position 54 DENV-2	A54E	Increased the fusion threshold to a higher pH Slight delay in viral replication in Vero cells	[91]
Envelope (E)	Amino acid position 280 in DENV-2	T280Y	Slight reduction in replication efficiency C6/36 cells compared to wild type	[91]
Envelope (E)	Amino acid positions 104 and 135 in DENV-2	G104S L135G	Fusion defective and non-infectious at 37 °C, but became infectious at 28 °C in mammalian cells Deficient in fusion activity in ADE conditions	[95]
Envelope (E)	Amino acid positions 144, 244, 261, 282 in DENV-2	H144A H244A H261A H282A	Displayed a 50% decrease in secretion of VLPs compared to the wild type	[97]
NS1	Amino acid positions 114, 115, 180, 301 in DENV-2	S114A W115A D180A T301A	Minor effects in viral replication Significant impairment in infectious virus production (~2.5log10 reduction compared to wild type)	[23]
NS2A	Amino acid position 84 in DENV-2	R84A/S	Abolished the formation of infectious virions but did not affect viral RNA synthesis	[121]
NS2A	Amino acid positions 11, 20, 100, 187, and 188 in DENV-2	G11A E20A E100A Q187A K188A	Produced 10^4^-fold less viral RNA than wild type Specifically impaired virion assembly without significantly affecting viral RNA synthesis	[125]
NS2A	Triple alanine mutations at amino acid positions 163–165, 178–180, and 184–186 in DENV-2	163NAW165 to AAA 178SPL180 to AAA 184SSQ186 to AAA	More than >100-fold reduction in viral titers Mild to moderate defects in infectious virus production Displayed a slight decrease in viral replication compared to the wild type	[126]
NS2A	Triple alanine mutations at amino acid positions 16–18, 21–23, 27–29, 50–52, and 56–58 in DENV-2	16ALF18 to AAA 21EML23 to AAA 27VGT29 to AAA 50FR52D to AAA 56VMV58 to AAA	Displayed > 1000-fold reduction in virus yield No formation of viral plaques, but exhibited wild type-like replication and infectious virus production levels	[127]
NS2B	Amino acid positions 50–89 in DENV-4	Deletion of 40 amino acids	Completely abolished autoproteolytic activity	[131]
NS3	Amino acid positions 129, 130, 133, 136, and 152 in DENV-2	D129I/K/R F130A/S G133A G136A N152A/Q	Significantly reduced NS3 protease activity	[134]
NS3	Amino acid position 283 in DENV-2	M283F	Reduced ATPase activity but increased helicase activity Exhibited temperature-sensitive phenotype, and at higher temperatures led to a reduction in RNA synthesis	[135]
NS3	Amino acid position 176 in DENV-4	P176G	Severe reduction of luciferase activity by 70%, indicating a decrease in viral replication efficiency compared to the wild type	[137]
NS3	Triple alanine mutations at amino acid positions 574–576	EEN to AAA	Slight delay in viral replication before exhibiting replication efficiency comparable to wild type Significantly decreased production of viral particles (>10,000-fold lower than wild type)	[138]
NS3	Amino acid position 290 in DENV-4	D290A	Resulted in a ~2-fold decrease in viral replication compared to wild type Generated small plaques	[139]
NS4A	Amino acid positions 53, 57, and 67 in DENV-2	E53A L57A G67A	Produced smaller plaques compared to wild type, except for E53A, which could only be visualised by immunostaining Decreased viral yields	[144]
NS4A	Amino acid positions 40 and 44 in DENV-2	A40G A44G	Demonstrated severe reduction in viral replication (<1000 PFU/mL virus) A40G formed smaller plaques compared to the wild type A44G formed plaques, similarly, resembling the wild type	[144]
NS4A	Amino acid position 41 in DENV-2	Y41F	Viable despite severely attenuated Exhibited RNA and viral replication kinetics (>1log reduction in viral RNA levels)	[146]
NS4B	Amino acid position 144 in DENV-2	N144A	Demonstrated 1000-fold reduction in virus yield compared to the wild type Produced slightly smaller plaques than the wild type	[149]
NS4B	Amino acid position 58 in DENV-2	N58Q	Slight reduction in viral replication and production efficiency Did not affect viral polyprotein translation and processing	[150]
NS4B	Amino acid position 62 in DENV-2	N62Q	Severely decreased viral replication and viral production efficiencies Did not affect viral polyprotein translation and processing	[150]
NS4B	Amino acid positions 144, 147, 149 in DENV-2	N144A V147A G149A	Severely attenuated viral replication compared to the wild type	[151]
NS4B	Amino acid position 116 in DENV-1 isolates from the 2013 Vietnam dengue epidemic	116V	Decreased virus growth in mammalian cells, but displays enhancement in virus growth in mosquito cells	[152]
NS5	Amino acid positions at 325 and 769 in DENV-2	R325A R769A	Exhibited wild type-like replication in vitro Delayed or impaired viral replication in cell culture	[161]
NS5	Double alanine mutations at amino acid positions 519 and 523 and 840 and 841 in DENV-2	R519A/K523A K840A/R841A	Exhibited wild type-like replication in vitro Delayed or impaired viral replication in cell culture	[161]
NS5	Double alanine mutations at amino acid positions 361 and 370 in DENV-2	R361A/K370A	40% reduction in RNA polymerase activity compared to the wild type Delayed replication phenotype in cell culture in vivo	[161]
NS5	Amino acid position 888 in DENV-2	R888K	Produced viable mutant viruses with a 90% decrease in infectivity compared to wild type Did not affect the localisation of DENV-2 NS5 in the nucleus	[163]
NS5	Amino acid position 264 in DENV-3	V264G	Increased flexibility of the NS5 linker Decreased in RNA polymerase activity (75% decrease compared to wild type) Slightly attenuated mutant viruses	[164]
3′ UTR	Nucleotide positions 10299, 10387, 10396, 10411, 10449, 10538, 10551, 10566, 10571, 10605 in DENV-2	10 nucleotide mutations G10299A, T10387C, T10396C, A10411G, C10449T, T10538C, C10551T G10566A, G10571A and G10605A	Reduced infectivity of the mutant virus Significantly lower translation efficiency	[173]
3′ UTR	Nucleotide deletions (Δ10,474–10,478) and (Δ10,562–10,566) in DENV-2	Deletion of 5 nucleotides per region	Severe reduction in viral replication Displayed a 60% reduction in viral translation	[175]

**Table 2:** Nucleotide changes in the DENV genome and the impact of these mutations in in vitro and in vivo studies.

Genome Region	Position in the Genome	Mutation(s)	Consequences of the Mutation	References
5′ UTR	Nucleotide 69 in DENV-2	A69ntT	Reduced the mortality rate of newborn ICR mice by 31.25% compared to wild type (84.37%)	[69]
Capsid (C)	Nucleotide (Δ42–59) deletion in DENV-2	Deletion of 18 nucleotides	Mutated viruses were highly attenuated in suckling mice Generated high levels of antibodies in adult mice	[75]
prM/M	Amino acid positions 39 in DENV-1 and 43 in DENV-4	H39R A43T	Reduced virus titers (~0.7–1.4log10) compared to wild type (~2.2–3log10) Demonstrated low levels of viraemia in rhesus monkeys	[81]
prM/M	Amino acid position 86 in DENV-2	H86R	Generated small plaques and low viral replication activity compared to the wild type Reduced intracellular expression of prM, E, and NS3 proteins in Vero cells No reduction in viral protein translation in C6/36 cells	[84]
Envelope (E)	Amino acids 324, 351, and 380 in DENV-1	I324V L351V I380V	Displayed lower infectivity than wild type in mammalian cells and BALB/c suckling mice Decreased viral titers in mutant virus-infected cells compared to wild type infected cells	[96]
NS3	Amino acid position 192 in DENV-4	D192N	Significant reduction in viral replication in the brains of suckling mice (>10,000-fold reduction compared to the wild type) Exhibited small plaques, temperature-sensitive phenotype in mammalian cells, and animal models Reduction of viraemia in SCID-Huh-7 mice (~3.6log10 PFU/mL reduction compared to the wild type DENV-4- infected SCID mice	[136]

## 19. Potential Solutions to Circumvent Antibody-Dependent Enhancement (ADE) Effect

Given that the ADE phenomenon is a massive setback in the development of live-attenuated dengue vaccine, researchers have been utilising various strategies with a single objective in mind, to reduce or completely abolish ADE. One promising approach to mitigate ADE is the introduction of targeted mutations into several key DENV proteins, which could modify non-neutralising antibody interactions, ablating ADE-linked epitopes, and even alter FcγR engagement. Several studies have indicated that the prM protein was associated with DENV ADE, highlighting the role of prM-specific monoclonal antibodies in enhancing DENV infection [177]. Following the first DENV infection, it has been observed that a considerable fraction of circulating antibodies was predominantly anti-prM antibodies [20]. These prM antibodies were reported to be highly cross-reactive against all four DENV serotypes and displayed poor neutralising capacity against heterotypic DENVs [178]. Additionally, prM antibodies have been demonstrated to enhance the infectivity of partially mature DENV particles [179]. A pr4 epitope (amino acid positions 19–34 of DENV/JEV) was suggested to markedly influence ADE [177]. Cui et al. (2021) constructed a chimeric DENV-2 virus by substituting the DENV pr4 gene with the corresponding JEV pr4 gene (Table 3) Reduced ADE effect and viral infectivity with the chimeric DENV-2 virus were observed in comparison to the wild type DENV-2 virus. Additionally, 16 alanine substitutions were individually introduced across the DENV-2 pr4 epitope, thereby generating pr4 variants, pr4.1 to pr4.16. Three pr variants, pr4.5 (L23A), pr4.6 (L24A), and pr4.7 (F25A), contained the respective alanine mutations that demonstrated a significant reduction of enhancement effect in DENV-2 infection in FcγR-bearing K562 cells compared to the wild type pr4, thus decreasing the occurrence of ADE.

Further characterisation of a DENV cross-reactive monoclonal antibody, 4D10, which poorly neutralised DENV but greatly enhanced infection of all four DENV serotypes, resulted in the identification of a novel enhancing epitope of 4D10 that was correspondingly mapped to the prM protein, specifically amino acid residues at positions 14–18 (VSRQE) [180]. The epitope of 4D10 mAb is highly conserved among DENV serotypes and was demonstrated to be immunogenic in BALB/c mice. Peptide PL10 containing the 4D10 epitope (IVSRQEKGKS) demonstrated high immunogenicity in BALB/c mice and was recognised as a DENV cross-reactive epitope. Antibodies generated against PL10 displayed weaker neutralising activity and broad cross-reactivity against DENV serotypes, leading to the enhancement of all DENV1-4 infections in infected K562 cells during ADE assays, similar to the 4D10 mAb. Further mutagenesis studies could be performed to determine if introducing mutations to this specific epitope identified in prM protein would eliminate or modify ADE effects, and this could have potentially significant implications for novel live-attenuated dengue vaccine development. Smith and colleagues (2016) isolated a panel of 25 naturally occurring human monoclonal antibodies that specifically targeted the DENV prM protein from DENV seropositive individuals or individuals that had been previously vaccinated with a live attenuated DENV-1 vaccine to elucidate the antigenic landscape of prM antibodies contributing to enhancement of replication [181]. An alanine scanning-based mutagenesis approach was utilised to map residues within the prM protein for antibody binding. Seven highly conserved residues (F1, L3, S5, E9, E18, L24, K26) among DENV serotypes were identified with >50% binding activity to the panel of mAbs. Among the prM residues, K26 was identified to be the most frequently involved residue, with 71% binding to the panel of mAbs. Thus, the research findings demonstrated that these isolated human mAbs recognised a single major antigenic site containing these seven residues on the prM protein, especially K26, and acknowledged the need for vaccine strategies that would minimise the induction of cross-reactive prM-specific antibodies to avoid ADE. As such, the reduced induction of prM-specific antibodies and the potential ablation of the immunodominant K26 residue would be the way forward toward the development of a novel DENV vaccine without ADE.

Apart from the prM protein, the E protein is a primary target in minimising ADE effect, as in the presence of cross-reactive non-neutralising anti-E antibodies, it showed an enhancement effect in viral replication by replicating to high titers in human monocytes in vitro [16,182]. During a primary DENV infection, antibodies are normally generated against the E protein [182]. However, in secondary DENV infection with a different DENV serotype, pre-existing non-neutralising antibodies might interact and bind to the E protein, but they were unable to neutralise DENV effectively. The main repertoire of antibodies in human serum predominantly targeted the fusion loop of the E-DII domain, and they were identified as cross-reactive but poorly neutralising [182,183]. Substitution mutations at three fusion loop residues (W101A, L107A, F108A) within individual E mutant proteins intriguingly led to nearly complete abolishment of their binding activities to highly cross-reactive anti-E antibodies, including the flavivirus-group reactive 4G2 mAb from the sera of DENV-infected patients (Table 4) [182]. Crill and colleagues (2012) developed a cross-reactivity reduced (CRR) DENV-1 DNA vaccine containing cross-reactive B cell epitopes associated with immune enhancement [184]. A total of five individual substitutions were introduced into the E-DII fusion loop containing immunodominant B cell epitopes (G106R, L107D) capable of inducing broadly cross-reactive non-neutralising antibodies and the E-DIII domain containing cross-reactive antigenic regions (K310D, E311K, P364Q). CRR-vaccinated mice produced significantly lower levels of antibodies recognising immunodominant, enhancing cross-reactive epitopes and exhibited reduced DENV-2 disease enhancement, with 68% survival upon heterologous DENV-2 challenge. These findings demonstrated that the incorporation of these amino acid substitutions to dampen the immunodominant epitopes in the CRR DENV-1 DNA vaccine could significantly reduce the risk of ADE and enhance a more serotype-specific protective immunity, potentially addressing a major challenge in dengue vaccine development. In the development of a novel VLP-based tetravalent dengue vaccine, the incorporation of an F108A mutation in the ultra-conserved E protein fusion loop region led to an increase in VLP production in mammalian cells [185]. The VLP vaccine elicited high levels of neutralising antibody responses against all four DENV serotypes in VLP vaccine-immunised mice and intriguingly demonstrated the absence of ADE with the lowest possible dilution of serum.

Two prM-specific mAbs, 1G6 and 5M22, identified from the earlier study by Smith et al. (2016), were found to recognise quaternary epitopes present on the surface of viral particles where both prM and E proteins commonly interact [181]. Mutagenesis studies identified five highly conserved residues within the E-DII fusion loop (W101, G102, N103, G104, G111) and one in the E-DIII (H242) of the E protein that were essential for the binding activity with 1G6 and 5M22 mAbs, indicating that these residues could contribute to ADE in severe dengue infections. The involvement of quaternary epitopes highlighted the need to highly consider both prM and E proteins in the development of a dengue vaccine. One of the most potent cross-neutralising antibodies capable of neutralising all four DENV serotypes, SIgN-3C, which specifically targeted a dimeric epitope in the fusion loop, was identified [186,187]. Alanine scanning mutagenesis and epitope mapping studies revealed that the SIgN-3C interacted with a small cluster of E protein residues in the E-DII domain (G100, W101) in one monomer and the E-DIII domain (K310, R323) in another monomer [186]. A “LALA” mutation within the Fc region of SIgN-3C, by substitution from leucine to alanine at positions 234 and 235 (L234A/L235A), abrogated binding to FcγR and severely reduced infection enhancement in K562 cells, indicating that the SIgN-3C LALA mutant prevented ADE. Thus, the identification of the highly conserved E residues recognised by SIgN-3C mAb could provide the design of a novel dengue vaccine with broad neutralisation capability and safety profiles with no ADE. Previously, Setthapramote et al. (2012) generated a human monoclonal antibody (HumAb) B3B9 with strong in vitro neutralising activity against all four DENV serotypes but showed ADE activity at sub-neutralising concentrations [188]. To eliminate ADE activity while retaining its neutralising capabilities, the N297 glycosylation site within the Fc region of the B3B9 antibody was modified via site-directed mutagenesis, thereby forming N297Q-B3B9 HumAb [189]. The N297Q-B3B9 antibody indeed retained its cross-neutralising capabilities against all DENV serotypes and intriguingly displayed complete reduction of ADE activity in K562 cells. Further epitope mapping via phage display approaches revealed that the modified antibody targeted a highly conserved region in the fusion loop of the E-DII domain located at amino acid positions from 107 to 111 (LFGKG) of the E protein.

**Table 4:** DENV E-specific residues associated with their corresponding mutation(s) within the epitopes recognised by DENV E-antibodies and their impact on ADE.

Residue	Mutation(s)	Epitope Sequence Recognised by DENV E-Antibodies	ADE Effects	References
Amino acid positions 101, 107, and 108 in DENV-1 to -3	W101A L107A F108A	E-DII fusion loop	Not validated in terms of mutations introduced into these residues would lead to modification of ADE	[182]
Amino acid positions 106, 107, 310, 311, 364 in DENV-2	G106R L107D K310D E311K P364Q	E-DII fusion loop E-DIII domain (amino acid positions 310,311 and 364)	Reduced risk of ADE	[184]
Amino acid position 108 in DENV-1 to -4	F108A	E-DII fusion loop	No ADE observed	[185]
Amino acid positions 101, 102, 103, 104, 111, 242 in DENV-1 to -4	W101A G102A N103A G104A G111A H242A	E-DII fusion loop E-DIII domain	Not validated in terms of mutations introduced into these identified residues would lead to modification of ADE	[181]
Amino acid positions 100, 101, 310 and 323 in DENV-1 to -4	G100A W101A K310A R323A	E-DII fusion loop E-DIII domain (amino acid positions 310 and 323)	Completely abrogated ADE	[186]
Amino acid positions 107–111 in DENV-2	NA	LFKGKG	NA	[189]

NA denotes not available.

Functional assays for evaluating dengue mutation:


**Assay**

**Function**

**Strengths**

**Limitations**

**References**
Plaque assay/focus-forming assayMeasures viral titers and evaluates replication efficiencyGold standard (plaque assay) for quantifying infectious lytic virions.Unable to differentiate between infectious and non-infectious particles Labor-intensive and time-consuming[190,191]Neutralisation assay (PRNT/FRNT)Assessing the capacity to elicit neutralising antibodies to neutralise the virusGold standard (PRNT) for neutralising antibodies in the assessment of the immunogenicityTime-consuming Variability in interpretation[192,193]Luciferase reporter assays/subgenomic repliconAssessing viral translation and replication independently via luciferase readoutRapid quantification and high sensitivity without the need to produce infectious virusesUnable to assess infectious particle production[23,194]RT-qPCRDetection of viral load Rapid quantification, high sensitivity, and specificity in viral detection Does not distinguish infectious from non-infectious RNA[195]ADE assayEvaluating antibody-enhanced infection in FcγR-bearing K562 cellsEssential for evaluating the risk of developing ADEMay not be able to fully replicate human-associated ADE in vivo[196,197,198]Animal Models (C57BL/6, AG129, BALB/c, SCID mice, rhesus monkey)Utilised to evaluate virulence, disease pathogenesis, vaccine efficacy, and safetyIn vivo models Does not fully recapitulate the disease in humans, but closely resembled dengue manifestations in humans[35,81,136,199]

## 20. Proposed Live-Attenuated Dengue Vaccine Candidate

**Figure 4:**
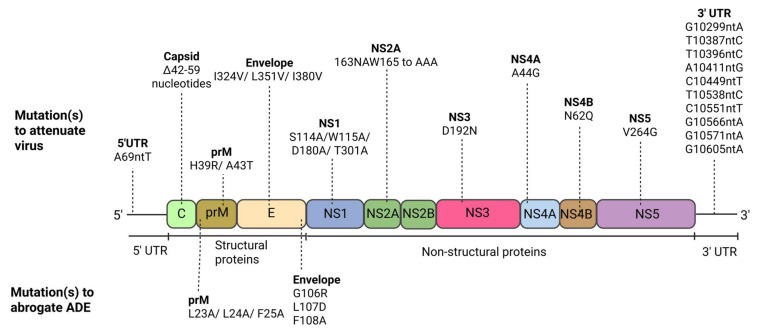
Proposed live-attenuated vaccine candidate against dengue. The proposed vaccine candidate harboured mutations associated with reduced replication competency and minimal antibody-dependent enhancement (ADE).

We selected ideal attenuating mutations across each gene indicated in the DENV genome, where they were selected for their ability to reduce replication competency without severely compromising the capacity of the attenuated virus to infect target cells and replicate in vaccinated hosts (Figure 4) [23,69,75,81,96,126,136,144,150,164,173,177,182,184,185]. Given that several mutagenesis studies have validated these attenuating mutations in both the in vitro and in vivo systems, they were prioritised over mutations that were only validated in vitro. These validated attenuating mutations have been validated in the 5′ UTR region, capsid, prM, envelope, and the NS3 genes [69,75,81,96,136]. For the remaining DENV genome regions, attenuating mutations were selected based on significantly reduced viral RNA replication and infectious viral particle production [23,126,144,150,164,173]. In the highly attenuated mutant viruses, they were able to elicit high levels of humoral antibodies and cellular immunity. Additionally, we also incorporated mutations that were demonstrated to reduce or abrogate ADE effects into the proposed vaccine candidate. DENV prM mutations (L23A/L224A/L25A) were selected as they were able to significantly decrease enhancement effects in DENV-2 infection, thus decreasing ADE in the process [177]. Located within the E-DII fusion loop region, three DENV E mutations (G106R/L107D/F108A) have previously been characterised to ablate fusion loop activity and impeded the generation of fusion-loop-specific, poorly neutralising antibodies known to be responsible for driving ADE [182,184,185].

## 21. Future Directions in the Development of Live-Attenuated Dengue Vaccines

As dengue continues to pose a significant public health threat globally, and the WHO reported approximately 7.6 million dengue cases in 2024 alone, there remains an urgent need to develop an effective dengue vaccine [7]. With more than half of the world’s population at risk of dengue infection due to their geographical locations, dengue places a huge burden on the current healthcare systems, and economies, and exerts a significant impact on human societies [200]. Since DENV has four serotypes that are genetically related but antigenically distinct, the development of a dengue vaccine that can effectively target all four DENV serotypes by eliciting equitable levels of protection remains a monumental task [201]. Achieving an ideal tetravalent protection against dengue is rather challenging, as immune responses to one DENV serotype may be more immunodominant than the other serotypes, leaving vaccinated individuals highly susceptible to developing severe dengue infections upon infection by a heterologous DENV serotype. Hence, the inability of a vaccine to elicit equitable levels of neutralising antibodies against all four DENV serotypes can lead to the induction of ADE [202].

The underlying issue was evident in vaccinees receiving the first licensed dengue vaccine, CYD-TDV, which demonstrated varying efficacies against the four serotypes, and there was an increased risk of severe dengue in seronegative individuals. A likely theory of the observed severe dengue infections may be potentially due to the utilisation of a yellow fever backbone (YFV-17D), which incorporates the prM and E structural genes of the four DENV serotypes [203]. Due to the absence of the DENV non-structural genes in the CYD-TDV, CYD-TDV vaccination results in the production of antibodies against YFV non-structural proteins instead of the DENV non-structural proteins [204]. These YFV-generated antibodies might interact and bind to DENV, but they were not involved in neutralising the virus. Consequently, these could lead to the development of ADE, due to the absence of effective neutralising antibodies. On the other hand, the second live-attenuated dengue vaccine, TAK-003 was shown to exhibit long-term efficacy against all DENV serotypes in dengue-seropositive individuals but could only protect against DENV-1 and DENV-2 in dengue-seronegative individuals in a long-term study [50,205]. Only the full DENV-2 genome was present in the TAK-003 vaccine backbone, which could explain the higher efficacy against DENV-2 [203]. Despite its demonstrated efficacy and safety in vaccinees, the efficacy risk against DENV-3 and DENV-4 in dengue-seronegative patients remains to be further established, prompting the WHO to only recommend the utilisation of TAK-003 in highly dengue-endemic countries [45]. The TAK-003 vaccine remains a highly recommended live-attenuated vaccine to confer protection against hospitalisation of dengue-infected patients, although it remains to be established if vaccine-induced enhancement of dengue disease caused by DENV-3 or DENV-4 could be present in TAK-003 vaccinees. TV003/TV005, a third live-attenuated dengue vaccine candidate, is composed of three attenuated full-length viruses and one chimeric virus, generated by swapping the DENV-4’s prM and E genes with those from DENV-2. It is currently in phase III clinical trials and has demonstrated high vaccine efficacies against DENV-1 and DENV-2, irrespective of dengue serostatus. However, vaccine efficacies remained undetermined against DENV-3 and DENV-4 serotypes due to the lack of cases observed for these two specific DENV serotypes. Also, the Butantan-DV, which is analogous but not identical to TV003, is currently undergoing phase III clinical trials and has recently demonstrated convincing safety and efficacy profiles against DENV-1 and DENV-2 serotypes only [63].

In this review, we summarised a considerable number of highly conserved molecular determinants of the DENV genome that are likely to be associated with reduced virulence observed in both in vitro and in vivo studies. As all the nucleotides and amino acid residues presented in this review have been demonstrated to be conserved across all DENV serotypes, mutating these nucleotides should be able to attenuate or reduce virulence in all four DENV serotypes. However, developing a live attenuated dengue vaccine using attenuating mutations to significantly reduce virulence requires a considerable balance between the immunogenicity, safety, and inherent stability demonstrated by the vaccine construct. Additionally, selecting the appropriate attenuating mutations in specific DENV genes must be considered to evaluate reduced replication competency and the ability of the live-attenuated vaccine to elicit a robust and protective immune response while minimising the risk of ADE. Therefore, introducing attenuating mutations distributed across each of the DENV genomes could potentially produce an ideal vaccine candidate with no reversion. Another likely scenario to produce an ideal vaccine candidate could be by retaining the backbone of each DENV serotype genome while incorporating the multiple mutations into each DENV serotype genome. This might provide a solution to circumvent antigenic interference and immunodominance between different serotypes.

Recent studies have identified several ADE-linked residues that were recognised by cross-reactive non-neutralising antibodies in humans. The presence of these antibodies could lead to the enhancement of DENV infection in FcγR-bearing K562 cells. Although the selected amino acid residues demonstrated minimal ADE or could prevent ADE, follow-up studies are needed to determine if these modified residues are structurally stable when combined with the selected attenuating mutations elsewhere in the genome to reduce DENV virulence. As such, there needs to be an optimal balance between attenuating the virus to reduce virulence whilst eliminating or significantly reducing ADE by modifying ADE-associated epitopes without compromising the reduction in replication. Halstead and colleagues (2010) indicated that highly specific antibodies that were directed against the DENV E-DIII domain instead of the full E protein are essential and could avoid the stimulation of the ADE phenomenon [206]. From this observation, a designed DENV vaccine with the E-DIII domain of the E protein could potentially elicit E-DIII-directed serotype-specific antibodies without the generation of cross-reactive antibodies to the E-DII fusion loop epitopes, and this could result in conferring protection against ADE [207]. Thus, the mutants with highly conserved mutations identified only in the in vitro studies would need to be further validated to determine their reduced virulence and ADE in future in vivo studies.

## 22. Conclusions

In this review, highly conserved mutations that could lead to the significant reduction of virulence and ADE were reported, and these mutations have been validated in animal models. A tetravalent live-attenuated vaccine candidate against dengue can be constructed carrying the multiple mutations associated with minimal virulence, and ADE should be further assessed for its efficacy and safety in pre-clinical studies. Developing an effective live-attenuated vaccine is a promising strategy in the fight against dengue in resource-poor and low-income countries [208]. Despite major challenges faced by live-attenuated vaccines, such as the need for balanced protection against all four DENV serotypes and the risk of developing ADE, the ultimate goal remains attainable through identifying appropriate mutations that could be validated in vivo.

## Figures and Tables

**Table 3:** DENV prM-specific residues with their corresponding mutation(s) within the epitopes recognised by DENV prM-antibodies and their impact on ADE.

Residue	Mutation(s)	Epitope Sequence Recognised by DENV prM-Antibodies	ADE Effects	References
Amino acid positions 23, 24, and 25 in DENV-2	L23A L24A F25A	KGKSLLFKTENGVNMC	Significantly reduced ADE	[177]
Amino acid positions 14–18 in DENV-1 to -4	NA	IVSRQEKGKS	Not validated in terms of introducing mutations to these identified residues to determine ADE	[180]
Amino acid positions 1, 3, 5, 9, 18, 24, and 26 in DENV-1 to -4	F1A L3A S5A E9A E18A L24A K26A	Predicted epitopes: FHLTTRNGEPHMI VSRQEKGKSLLFK	Not validated in terms of introducing mutations to these identified residues to determine ADE	[181]

NA denotes not available.

## Data Availability

Not applicable.
